# Light‐Controlled Mammalian Cells and Their Therapeutic Applications in Synthetic Biology

**DOI:** 10.1002/advs.201800952

**Published:** 2018-09-30

**Authors:** Maysam Mansouri, Tobias Strittmatter, Martin Fussenegger

**Affiliations:** ^1^ Department of Biosystems Science and Engineering ETH Zurich Mattenstrasse 26 CH‐4058 Basel Switzerland; ^2^ Faculty of Science University of Basel Mattenstrasse 26 CH‐4058 Basel Switzerland

**Keywords:** cell engineering, mammalian cells, optogenetics, synthetic biology

## Abstract

The ability to remote control the expression of therapeutic genes in mammalian cells in order to treat disease is a central goal of synthetic biology‐inspired therapeutic strategies. Furthermore, optogenetics, a combination of light and genetic sciences, provides an unprecedented ability to use light for precise control of various cellular activities with high spatiotemporal resolution. Recent work to combine optogenetics and therapeutic synthetic biology has led to the engineering of light‐controllable designer cells, whose behavior can be regulated precisely and noninvasively. This Review focuses mainly on non‐neural optogenetic systems, which are often used in synthetic biology, and their applications in genetic programing of mammalian cells. Here, a brief overview of the optogenetic tool kit that is available to build light‐sensitive mammalian cells is provided. Then, recently developed strategies for the control of designer cells with specific biological functions are summarized. Recent translational applications of optogenetically engineered cells are also highlighted, ranging from in vitro basic research to in vivo light‐controlled gene therapy. Finally, current bottlenecks, possible solutions, and future prospects for optogenetics in synthetic biology are discussed.

## 1 Introduction

Light is strongly linked to a variety of processes of life.^1^ Sunlight is a vital source of energy for all living organisms on earth, and autotrophic organisms provide us with oxygen and chemical energy through the light‐dependent process of photosynthesis.^2^ Light is also essential for activation of important signaling pathways, e.g., phototransduction in the eye^3^ or regulation of the night–day cycle in plants.^4^ Molecularbiology approaches have identified many light‐sensitive proteins in bacteria, yeast, plant, and animal cells, and these can be used to introduce light sensitivity into otherwise insensitive cells from all kingdoms of life.^5^, ^6^ These light‐sensitive proteins can be genetically targeted to and expressed in any cell type, but have been most widely employed to manipulate neural activity.^7^, ^8^, ^9^, ^10^ This technique is called optogenetics, and combines the use of light and genetics to control cellular activity and behavior with high spatiotemporal resolution.^8^, ^11^

On the other hand, synthetic biology is the science of reassembling standardized biological components in a systematic and rational manner to create and engineer biological designer devices, systems, and organisms with novel and useful functions.^12^, ^13^, ^14^ During the past decade, mammalian synthetic biology has progressed from simple control switches to complex gene networks for biomedical applications in animal models, including T‐cell therapy^15^ and treatment of gouty arthritis,^16^ obesity,^17^ psoriasis,^18^ diabetes,^19^, ^20^, ^21^, ^22^ and many other conditions.^23^, ^24^, ^25^ Optogenetic methods offer many advantages, such as highly precise, efficient, and simple control of biological functions with rapid reversibility and reduced side effects, and the combination of these methods with synthetic biology has already enabled the engineering of light‐controlled designer cells that are useful both for basic research and for human gene and cell therapy in a noninvasive and precise way.

In this Review, we first point out the advantages of optogenetic methods over other commonly used inducer systems. Next, the state‐of‐the‐art optogenetic tool kit for mammalian cells is described. Then, we outline different optogenetic approaches that have been used to control biological functions in light‐sensitive engineered designer cells. Finally, we summarize recent applications and advances in the use of light‐controlled therapeutic cells in synthetic biology and biomedicine.

## 2 Potential of Optogenetics for Programing Engineered Mammalian Cells

Cells are constantly sensing and responding to extracellular stimuli in their environment.^26^ Many input signals, such as inducer molecules,^27^ temperature,^28^ pH,^29^ and electromagnetic radiation,^30^ including light, can activate or inhibit specific proteins or signaling pathways.^31^ Among them, chemically induced systems are routine tools in basic research and synthetic biology alike. However, chemical systems usually suffer from complex pharmacokinetics, including widespread and nonspecific induction, as well as difficulties in removing the inducer after a suitable induction period.^32^ These limitations restrict the precision with which the user can control the degree and spatial extent of activation, as well as the reversibility of a given output.

In contrast, optogenetic tools offer a promising opportunity for controlling cellular behavior with rapid responses, good reversibility and high spatiotemporal resolution.^33^ Optogenetics can efficiently induce a well‐defined subset within a population of cells, or even a single cell, as well as parts of an entire signaling network, with a desired frequency and duration of stimulation.

Genetically encoded tools for optogenetics were introduced as recently as 2005, when Boyden et al.^9^ recorded electrical activity in illuminated neural cells transfected with channelrhodopsin. This microbial rhodopsin had previously been functionally expressed in *Drosophila* 34 and mammalian cells.^35^ Since then, optogenetics has transformed experimental neurobiology by enabling the regulation of neuroelectric activities by light in vivo. Nowadays, these tools are gaining wide acceptance in biomedical research, as well as in the field of synthetic biology.

Rendering mammalian cells sensitive to light requires two elements: light‐sensing proteins and a light‐responsive module that is activated by the sensing part. Light sensing can be mediated by membrane‐bound photoreceptors or cytoplasmic photoactivatable proteins. Photoreceptors are usually localized on the cell surface and initiate signal transduction either by triggering subsequent signaling cascades or by changing the action potential across the plasma membrane. Cytoplasmic photoactivatable proteins, however, are not dependent on localization to the plasma membrane, and change their conformation upon absorbing energy from light. This structural rearrangement modulates intra‐/interprotein interactions, which can then initiate signal transduction.^26^, ^32^, ^36^

## 3 The Optogenetic Toolbox

**Figure 1:**
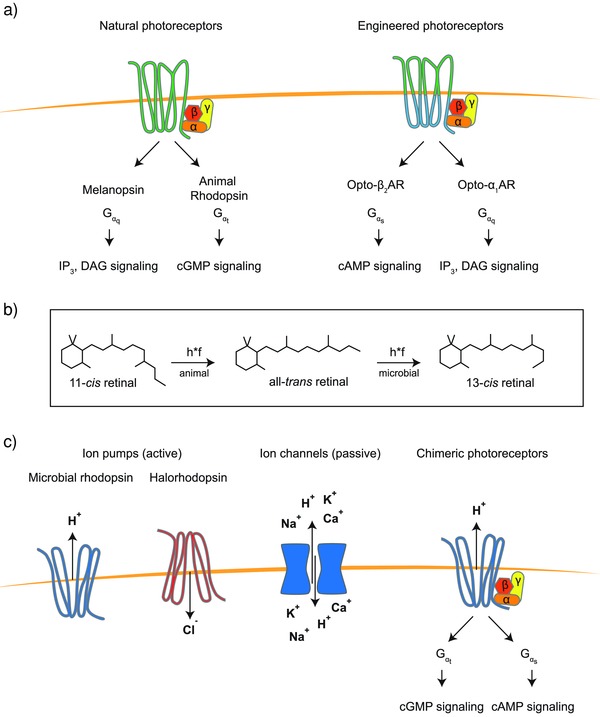
Opsin‐based photoreceptors: a) natural G‐protein‐coupled (GPC) photoreceptors such as melanopsin or animal rhodopsin signal through endogenous pathways. The specificity of signaling is determined by the different alpha subunits in the trimeric G‐protein complex. Gαq subunit–dependent signaling is linked to increased levels of inositol‐3‐phosphate (IP3) and diacylglycerol (DAG), whereas Gαt signaling mainly results in lowered cGMP levels that trigger subsequent signal amplification steps in photoreceptor cells of the mammalian retina. The intracellular binding loops for the trimeric G‐protein were exchanged to reroute Gαt to Gαs or Gαq signaling in engineered versions of bovine rhodopsin. These membrane‐bound GPC photoreceptors are retinal‐dependent. b) Exposure to light leads to activation of the bound retinal conformer and a subsequent change in conformation that is translated to structural changes in the apoprotein. While microbes incorporate mainly all‐*trans*‐retinal that is activated to 13‐*cis*‐retinal in melanopsin, mammals use 11‐*cis*‐retinal that is transformed into its all‐*trans*‐conformer upon illumination. c) Besides GPCRs, there are various GPCR‐like photoreceptors with high structural and amino‐acid sequence homology that do not act via G‐proteins. These receptors enable transport of protons or ions across the membrane. Examples of this class of receptors are the light‐driven proton pumps microbial rhodopsin and halorhodopsin. Photoswitchable ion channels enable passive transport of cations across the membrane. Chimeric photoreceptors combine features of GPCRs and microbial rhodopsin and can trigger both G‐protein‐dependent signaling and changes in membrane potential.

Photosensors, including opsin‐based photoreceptors and nonopsin photoreceptors (photoactivatable proteins), are at the core of the optogenetic tool kit.^37^ The most powerful and widely used photoreceptor systems in neural optogenetics (ion flux–based optogenetics) come from the opsin photoreceptor family.^6^, ^11^ Opsins are light‐sensitive transmembrane proteins that are found in a variety of organisms ranging from microbes to primates (**Figure** **1**). They can be categorized into two major classes: microbial opsins (type I) and invertebrate/vertebrate opsins (type II).^38^ Type I opsins are of great interest in the field of neural optogenetics, where they are used to control the function of neurons, because they are easier to engineer and express in mammalian cells (as a single‐component protein), and have faster kinetics than type II opsins.^6^ In addition to type I and II opsins, there are some engineered variants of opsins, which usually combine features of type I and type II opsins.^39^ Non‐neural optogenetics (i.e., non‐ion flux–based optogenetics) usually employs nonopsin photoactivatable proteins or gene switches.^40^ This type of optogenetics is the focus of this Review paper. Additional information about non‐ion flux–based optogenetics is available at www.optobase.org, which is a comprehensive online platform for optogenetics.

In addition to their applications in neural optogenetics, both opsin‐based photoreceptors and photoactivatable proteins have been used in synthetic biology,^32^, ^40^ as will be discussed in detail hereafter.

In order to respond to light as an environmental stimulus, most photosensors harbor an organic chromophore or cofactor. These small molecules bind to the photosensor and determine its spectral sensitivity and photochemistry. Following light absorption, changes in electron density occur within the chromophore, leading to a change of its structure, which in turn causes alterations in the surrounding protein scaffold of the photosensor. Ultimately, these modifications convert the photosensor into its active state, triggering downstream signaling pathways specific to that sensor.^41^

### 3.1 Vertebrate and Invertebrate Opsins

Type II opsins are found in animal and invertebrate cells and are primarily used for vision and for modulating circadian rhythms.^42^ These photoreceptors consist of a protein moiety (opsin) and a nonprotein moiety, which is often a vitamin A‐derived retinal chromophore.^43^ Opsins are G‐protein‐coupled receptors (GPCRs) (Figure 1a), which can initiate a signaling cascade upon activation by light.^38^ So far, more than a thousand animal opsins have been identified, and they can be divided into seven subfamilies.^42^ Although opsins have a seven‐transmembrane structure similar to that of other GPCRs, they are distinguished by a lysine residue in the seventh helix that serves as the binding site for the cofactor retinal.^39^, ^42^ Melanopsin, a representative member of animal opsins, is endogenously expressed in intrinsically photosensitive retinal ganglion cells (ipRGCs) of the inner retina.^44^ It plays a crucial role in the adaptation of mammals to different light intensities, as well as in the circadian timing system.^45^

During signal transduction, exposure to light induces isomerization of the retinal chromophore from 11‐*cis*‐retinal to all‐*trans*‐retinal, which subsequently changes the conformation of the melanopsin receptor^38^ (Figure 1b). These structural changes in the receptor activate the heterotrimeric G‐protein, causing dissociation of the Gαq subunit from the dimeric G*βγ* complex. The Gαq subunit further activates phospholipase C, which can initiate calcium‐dependent signaling within mammalian cells.^31^, ^36^

One major limitation in the use of melanopsin is its dependency on a steady supply of 11‐*cis*‐retinal for in vitro experiments. However, bistable invertebrate opsins are able to recover the *cis*‐isoform of retinal from the *trans*‐isoform, thereby bypassing the inherent bleaching issues of mammalian opsins. For example, an engineered version of *Carybdea rastonii* opsin, JellyOp, has been used modulate cyclic adenosine monophosphate (cAMP) levels when orthogonally expressed in mammalian cells.^46^

### 3.2 Microbial Opsins

Despite the lack of clear homology between the amino acid sequences of animal and microbial opsins, both receptor types are composed of seven‐transmembrane (TM) domains and both use retinal as a chromophore.^39^, ^42^ However, microbial opsins show little or no G‐protein activation^47^, ^48^ and unlike their mammalian counterparts, microbial opsins (type I) isomerize all‐*trans*‐retinal to 13‐*cis*‐retinal upon illumination (Figure 1b); this reverts to all‐*trans*‐retinal within the receptor during an inactive recovery phase. This cyclic process is referred to as a “photocycle” and consists of different intermediate states with the last step of the cycle replenishing the substrate of the first reaction, thus making the reaction fast and reversible.^49^ This is a distinctive feature of microbial opsins, since the corresponding process in vertebrate opsin (type II) is unidirectional or bidirectional, but not a cycle.^6^

Activated opsins of the type I family can function in various settings, such as light‐driven pumps, light‐gated channels, photosensors, and light‐activated enzymes.^43^, ^50^, ^51^, ^52^ Consequently,type I opsins have been widely used in neurobiology as optogenetic tools, as has been extensively reviewed elsewhere.^5^, ^6^, ^11^

Nevertheless, we would like to highlight the first identified microbial type I rhodopsin, bacteriorhodopsin (BR), which is a light‐driven outward H^+^ pump^53^, ^54^ (Figure 1c). So far, a number of microbial proton pumps (e.g., proteorhodopsin,^55^ delta‐rhodopsin,^56^ xenorhodopsin^57^) have been used in synthetic biology to pump protons in‐/outward across the plasma membrane of cells in response to light. By taking advantage of the delta‐rhodopsin H^+^ pump, Hara et al. generated a light‐driven proton motive force in the inner membrane of mitochondria in Chinese hamster ovary (CHO) cells, as well as neuroblastoma cells.^56^ These synthetic photoenergetic mitochondria provide an excellent example of the use of optogenetics to control the behavior of a well‐defined organelle inside mammalian cells.

Turning to proton pumps, the family of type I opsins includes ion channels that are also used in the field of optogenetics (Figure 1c). Channelrhodopsin‐2 (ChR2), for instance, is a light‐gated, nonspecific cation channel, which allows the passage of cations across the plasma membrane, leading to depolarization of the cell.^35^ Kushibiki et al. developed a light‐dependent circuit for insulin secretion in mouse pancreatic β‐cells by functionally expressing ChR2 in β‐cells. They demonstrated that blue light–mediated activation of ChR2 induces Ca^2+^ release from intracellular stores (e.g., endoplasmic reticulum). This leads to Ca^2+^ influx in engineered mouse pancreatic β‐cells, which in turn triggers the natural signaling cascade and can be used to regulate blood glucose homeostasis both in vitro and in vivo.^58^

### 3.3 Nonopsin Photoactivatable Proteins

**Figure 2:**
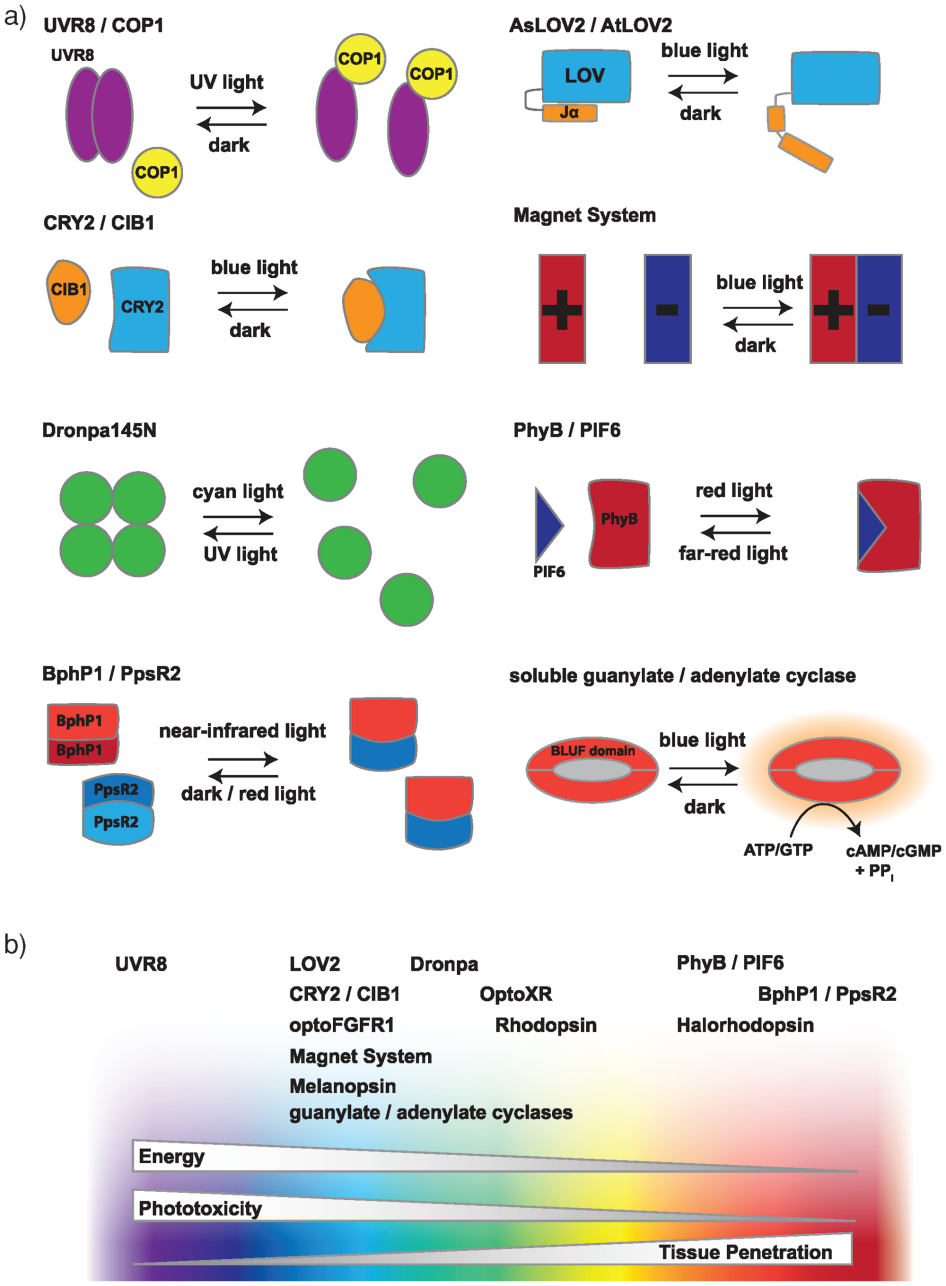
Nonopsin photoactivatable proteins: a) naturally occurring and engineered photoactivatable proteins can be activated by light of different wavelengths ranging from ultraviolet to infrared. Different mechanisms of activation are known: association, dissociation, uncaging, or unhinging as well as direct photoactivation of enzymes. For example, UV‐B light triggers a stress response in plants which is mediated by dimerization of UVR8 proteins. LOV domains from *As*LOV2 and *At*LOV2 are stimulated by blue light, causing the then‐unhinged C‐terminal alpha helix Jα to bend outward, activating a kinase domain in the natural protein. The CRY2 and CIB1 pair mediates responsiveness to blue light in plants via a blue light–induced heterodimerization mechanism. CRY2 also has a tendency to cluster into homo‐oligomers upon activation. In the Magnet system, engineered variants of the VVD domains are either positively or negatively charged. Upon exposure to blue light, they only bind to each other, but do not form homodimers. The Dronpa145N mutant of the fluorescent protein Dronpa was found to form homotetramers in the dark that dissociate on exposure to cyan light, just like the wild‐type protein, but at lower concentrations. UV light reverses this reaction. The photoreceptor protein PhyB binds to its partner PIF6. Far‐red light–induced dissociation of the complex reverses this interaction. The bacteriophytochrome BphP1 forms homodimers in the dark, but forms heterodimers with PpsR2 upon exposure to near‐infrared light. This transition is reversible and the backward reaction can be boosted by exposure to red light. Soluble guanylate cyclases are a class of photoactivatable enzymes that are activated by blue light through their BLUF (blue light sensor using FAD) domain. b) Photosensors can be activated by a specific wavelength in the UV–vis spectrum. While shorter wavelengths have higher energy, they also show greater phototoxicity, damaging DNA and perturbing pathways that involve chromophore‐binding proteins. Additionally, tissue penetration is limited for blue light compared to red light. In the retina as well as in many microorganisms, there are opsins for every section of the visible spectrum. However, most light‐inducible systems available for molecular biology are activated exclusively in the blue and green ranges.

Nonopsin photoactivatable proteins are widely used to optogenetically control intracellular signal transduction^26^ and have frequently been applied in the field of synthetic biology.^32^, ^40^ Photosensitive proteins are mostly based on proteins from bacteria, plants, or fluorescent proteins. Bacterial photosensitive proteins such as bacteriophytochrome P1 (BphP1), blue light–utilizing flavin adenine dinucleotide (BLUF), and bacterial cyclase (e.g., *Beggiatoa*‐photoactivated adenylyl cyclase (bPAC)^59^ and bacterial diguanylate cyclase (DGCL),^60^) as well as plant‐based photosensors such as the cryptochromes (i.e., cryptochrome 2 (CRY2)), UV‐B resistance 8 (UVR8), and light, oxygen, and voltage (LOV) domains, are well‐known in optical–synthetic biology. Alternatively, Dronpa is also available as a photoactivatable fluorescent protein (**Figure** **2**a).^61^

The LOV domain consists only of a small domain that mainly uses flavin mononucleotide (FMN) as a chromophore. Additionally, there are some variants of LOV domains (e.g., Vivid (VVD)^62^ and NcWC1^63^) that use flavin adenine dinucleotide (FAD) as a cofactor. Both the cofactors are ubiquitously produced by mammalian cells, and do not need to be provided externally.^26^, ^61^ Blue light activates the *Arabidopsis sativa* LOV2 (*As*LOV2) or *Arabidopsis thaliana* LOV2 (*At*LOV2) domain by removing the autoinhibitory effect of the C‐terminal helix Jα on the LOV core domain through the introduction of a twist in this helix. Based on this mechanism, researchers have developed several strategies to control the activity of an effector protein that is directly fused either to the N‐terminal of the LOV domain or to the C‐terminal of the Jα helix. In the resulting construct, the effector protein remains suppressed in the dark, while exposure to blue light leads to its activation.^64^

CRY2 is a FAD‐containing photoreceptor from *A. thaliana* that either binds to its native binding partner cryptochrome‐interacting basic helix–loop–helix 1 (CIB1) or homo‐oligomerizes with another CRY2. Both the configurations are activatable by blue light.^61^, ^65^, ^66^, ^67^ Providing another mode of action, the FAD chromophore in the photolyze homology region (PHR) domain of CRY2 can be reduced or oxidized in the presence or absence of light, respectively. Subsequently, the light‐induced reduction/oxidization triggers conformational changes in the domain that can be translated into different binding behaviors of the protein.

Phytochrome B (PhyB) is a plant photosensor that can be activated by red light and inactivated by infrared light.^26^, ^61^ The chromophore of PhyB is phycocyanobilin (PCB), which must be provided externally or by introducing the necessary enzymes for its synthesis, since it is not naturally produced in mammalian cells.^68^ Upon exposure to red light, PhyB changes conformation and binds to its partner phytochrome interacting factor (PIF). Interestingly, this interaction can be blocked again by inactivation of PhyB upon exposure to infrared light.^68^

A recently developed light‐switchable transgene system based on BphP1–PpsR2 or BphP1–QPAS1 interaction can be activated and inactivated by light in the near‐infrared region (740–780 nm) and red light (660 nm), respectively.^69^, ^70^, ^71^ BphP1 is a bacterial phytochrome and uses biliverdin, which is abundant in eukaryotic cells, as a chromophore.

UVR8 is another photoactivatable plant protein; it requires no cofactor and absorbs UV‐B light via a tryptophan residue.^71^ In the dark, UVR8 forms a homodimer, which dissociates and binds to constitutively photomorphogenic 1 (COP1) upon exposure to UV light.^72^

Light‐controlled enzymes (cyclases) convert adenosine triphosphate (ATP) or guanosine triphosphate (GTP) to the second messenger molecules, cAMP or 3′,5′‐cyclic guanosine monophosphate (cGMP), and activate special pathways.^59^, ^60^

Dronpa (“Dron” is Japanese for “vanish” and “pa” for “photoactivatable”) is a monomeric fluorescent protein (FP) that undergoes transition between fluorescent and dark states upon stimulation with cyan light (≈500 nm, for dark conversion) and violet light (≈400 nm, for reversion to fluorescent state). This FP has no cofactor, and uses a post‐translationally modified and extended tryptophan for light absorption.^73^ A mutated form of Dronpa, called Dronpa145N (discussed in more detail in the next part), forms homotetramers that can be dissociated into monomers with cyan light and retetramerized with violet light.^73^

A summary of the features of the proteins described above is provided in **Table** **1**.

**Table 1:** Properties of common photosensors used in light‐controlled mammalian cells

Light	Photosensor	Partner	Cofactor	Availability of cofactor in mammalian cells	Activation/inactivation [nm]	Origin	Mechanism of action in optogenetic system	Applications (example)	Ref.
UV	UVR8	–/COP1	Trp	Yes	300/Dark	*Arabidopsis thaliana*	Dimerization/dissociation	Protein secretion	78
Cyan/violet	Dronpa145N	–	Cys–Tyr–Gly	Yes	490/390	GFP variant	Homodimerization/homotetramerization	Membrane recruitment	63
Blue	LOV2 CRY2 Melanopsin	– CRY2/CIB1	FMN FAD 11‐*cis*‐Retinal	Yes Yes Yes	450/Dark 450/Dark 470/Dark	*Avena sativa* *A. thaliana* Human	Uncaging Heterodimerization/oligomerization Ca^2+^ release from intracellular stores and NFAT dephosphorylation	Protein degradation Genome editing Gene expression	79 96 21
Green	MRa) OptoXR	– –	all‐*trans*‐Retinalb) all‐*cis*‐Retinalb)	No –	572/Dark 504/Dark	Bacteria, archaea, fungi chimeric	Proton pump Ca^2+^ release from intracellular stores or cAMP synthesis	Light‐powered mitochondria Gene expression	51 64
Red/far‐red	PhyB	PIF6	PCBb)	No	650/750	*A.s thaliana*	Heterodimerization/dissociation	Membrane recruitment and cell signaling	80, 81
Near‐infrared	BphP1	PpsR2	BV	Yes	750/650 or Dark	*Rhodopseudomonas palustris*	Heterodimerization/dissociation	Gene expression	61, 121

^a)^ Microbial rhodopsin (e.g., delta‐rhodopsin)

^b)^ These cofactors must be provided to mammalian cells externally.

### 3.4 Chimeric and Customized Photosensitive Proteins

One approach to optimize light‐induced reactions in mammalian cells is photosensor engineering. OptoXRs, which are chimeric proteins consisting of bovine rhodopsin (Rh) in which the cytoplasmic loops are replaced with those of the Gs‐coupled hamster β_2_‐adrenergic receptor (β_2_‐AR) or Gq‐coupled human α_1_‐adrenergic receptor (α_1_‐AR), show light‐dependent activation of the adenylyl cyclase (Gs) or phospholipase C (Gq) pathways, respectively (Figure 1a).^74^ Another chimeric animal opsin receptor is Opto‐mGluR6 (metabotropic glutamate receptor 6), which was generated by combining the light‐sensing domain of melanopsin with the intracellular domain of mGluR6. This engineered photoreceptor activates a native signaling pathway within retinal cells, and was able to reliably restore vision in blind mice.^75^

Native microbial rhodopsins do not activate the G‐protein signaling pathway, but initiate transmembrane ion transport instead, or act as histidine kinases. However, Sasaki et al. generated a chimeric proton pump based on microbial rhodopsin, which was able to activate Gt‐coupled protein (transducin) in parallel with proton pumping (Figure 1c). Analogously to the engineering of the OptoXR system, they designed their chimeric photoreceptor by replacing the cytoplasmic loop of *Gleobacter* rhodopsin (GR) with intracellular loops of bovine rhodopsin.^47^ In addition, Yoshida et al. reported a chimeric microbial rhodopsin with Gs‐coupled protein activation ability through incorporation of the cytoplasmic loop of β_2_‐AR).^48^ These chimeric microbial rhodopsins can act as bifunctional tools in synthetic biology, since they can simultaneously hyperpolarize the membrane and initiate G‐protein signaling within the cell.

**Figure 3:**
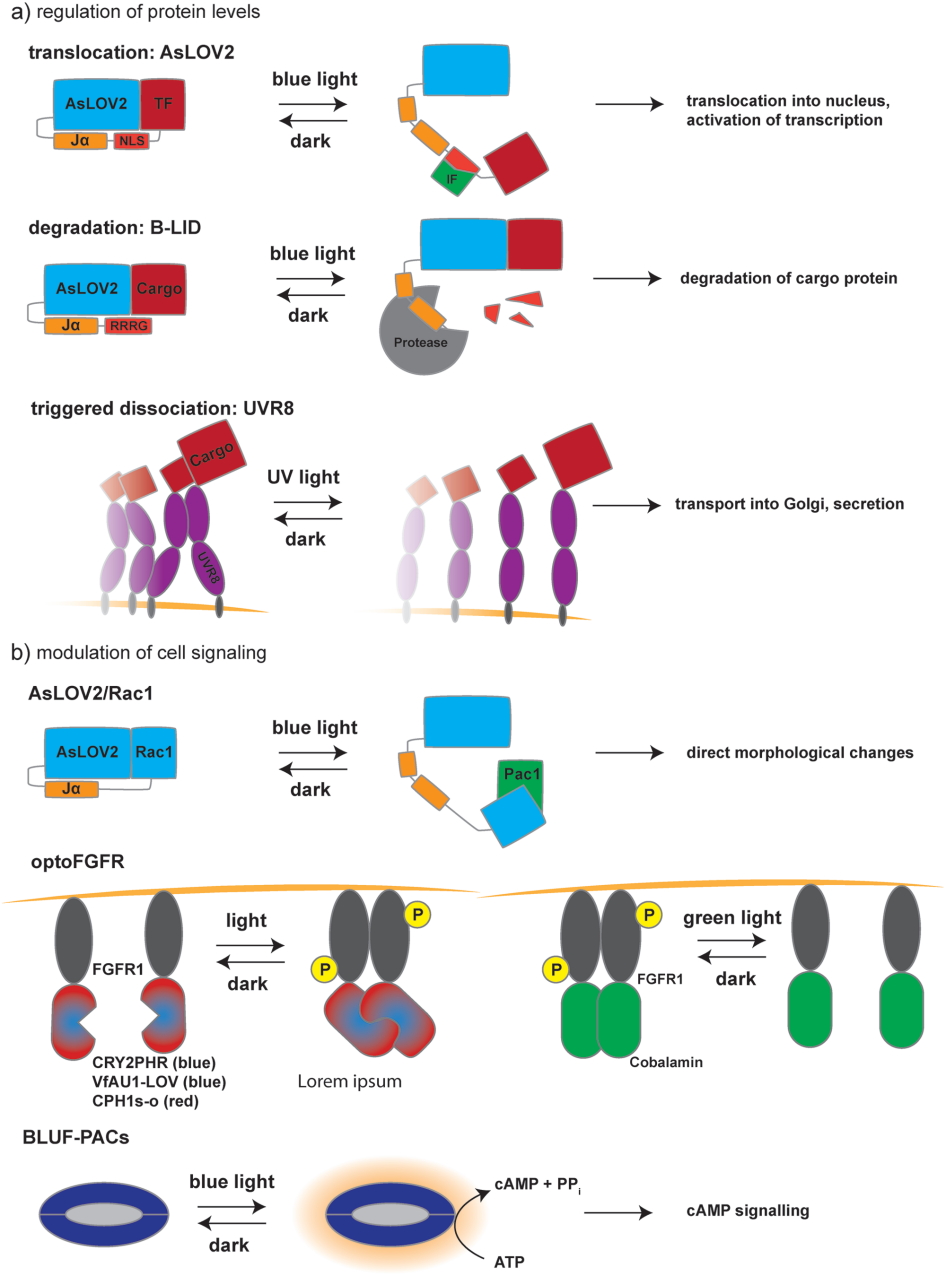
Applications for gene regulation and cell signaling: a) to modulate protein levels and localization directly and noninvasively with light, *As*LOV2 can be fused to a transcription factor linked via a nuclear localization signal. This construct unfolds upon exposure to blue light, making the NLS accessible to import factors that mediate translocation into the nucleus, where the transcription factor (TF) can act on its promoter to increase gene expression. In another strategy, *As*LOV2 is used as a photoswitchable degradation tag for a N‐terminally fused cargo protein. Again, blue light–induced unhinging is used to reveal the C‐terminal tetrapeptide degradation tag RRRG. Subsequently, the entire protein is degraded by proteases. In order to build a secretion system that can be precharged and timed, dimers of UVR8 were fused to a cargo protein targeted for secretion and tethered to the ER membrane. In the dark, the UVR8 tandem dimers form large clusters that effectively block transport from the ER to the Golgi. Upon exposure to UV light, these homo‐oligomers dissociate, allowing transport to the Golgi. b) In addition to regulating protein levels, photosensitive protein switches can be used to directly interfere with endogenous signaling pathways. To this end, *As*LOV2 was fused to Rac1, masking the binding site of Rac1. Upon light induction, the cognate binding partner Pac1 interacts with Rac1 and the respective signaling cascade is triggered, leading to morphological changes in the cell with high spatiotemporal resolution. The OptoFGFR system was used to build a whole array of inducible switches for the mitogen‐activated protein kinase/extracellular signal‐regulated kinase (MAPK/ERK) pathway by fusing engineered CRY2PHR, VfAU1–LOV, CPH1s‐o, or the cobalamin binding domain of *Thermus thermophilus* (*Tt*CBD or CarH) to the intracellular tyrosine kinase domain of the FGFR1 receptor of human or mouse. The resulting chimeric receptors are sensitive to blue, green, and red light, resulting in an ON‐type switch, except for the *Tt*CBD‐based receptor which showed OFF‐switch behavior. In another example, overexpression of BLUF domain–coupled photoactivatable guanylate cyclases (BLUF–PACs) in mammalian cells was used to modulate second messenger levels (e.g., cAMP) in response to blue light.

Engineering of photoactivatable gene switches can also improve the photoinduction ability of native receptors. For example, Kim et al. constructed a customized OptoFGFR1 by fusing the cytoplasmic tyrosine kinase domain (TDK) of human fibroblast growth factor receptor 1 (FGFR1) to CRY2PHR (a truncated version of wild‐type CRY2) (**Figure** **3**b).^76^ Here, activation of the chimeric receptor depends on light‐induced homodimerization of the CRY2PHR–TDK fusion construct.

It has also been shown that a mutation in the CRY2 moiety (the E409G mutant, named CRY2olig) can significantly improve the characteristic photoinduced clustering effect (homo‐oligomerization) of this receptor.^77^ Similarly, CRY2clust is an efficient CRY2‐based oligomerization system carrying a short peptide at the C‐terminus of the CRY2PHR domain; this serves to promote homo‐oligomerization of the protein upon exposure to blue light.^78^

Kawano et al. created the Magnet system based on the fungal photoreceptor VVD by substitution of wild‐type amino acids within the original VVD domain (a member of the LOV family) with either a positively or negatively charged amino acid, yielding pMag and nMag, respectively (Figure 2a). These pairs are engineered to recognize each other through electrostatic interactions, thus preventing unwanted homodimerization and selectively inducing heterodimerization in response to blue light.^79^ Intriguingly, the VVD domain is one of the smallest photosensors that uses the abundant FAD cofactor, and is thus a highly flexible tool for blue‐light optogenetics in mammalian cells.

Also, a tetrameric variant of Dronpa with a mutation at position 145 (K145N), denoted Dronpa145N, forms homodimers or homotetramers, which dissociate into monomers upon exposure to cyan light and reassociate upon exposure to violet light.^74^, ^80^ This mechanism can be used in a variety of applications in the field of synthetic biology (Figure 2a).

Engineered light‐sensitive receptors that can initiate certain pathways in mammalian cells have been successfully developed by fusion of photoreceptors to intracellular parts of the respective receptors. For example, fusion of CPH1s‐o^81^ or VfAU1–LOV^63^ to the a C‐terminal fragment of the FGF receptor‐1 makes it sensitive to red (660 nm) and blue (470 nm) light, respectively. These photoreceptor domains induce dimerization and finally activation of the receptors upon illumination. With the same strategy, but a different output, FGFR1 has been rendered sensitive to green light (545 nm) by fusion to cobalamin (vitamin B12)‐binding domains of bacterial *Thermus thermophilus* CarH transcription factors (*Tt*CBD or CarH).^82^ Here, green light induces receptor dissociation and inactivation, whereas the receptors stay active in the dark (Figure 3b).

## 4 General Optogenetic Strategies

In addition to the classical receptor‐based approaches, various other optogenetic strategies are available. Examples include uncaging, heterodimerization, and homodimerization/‐oligomerization, as well as association and dissociation^26^, ^61^, ^83^ (Figure 3).

Uncaging induces conformational changes in the structure of photoactivatable proteins, and can release inhibition of a protein fused to the photosensitive module or expose an otherwise concealed domain. The released protein/peptide can then interact with an effector protein or translocate, e.g., to the plasma membrane or the nucleus.^37^ For example, fusion of *As*LOV2 to Ras‐related C3 botulinum toxin substrate 1 (Rac1)^84^ or nuclear localization signal (NLS)‐tagged protein^85^, ^86^ resulted in cell movement and nuclear translocation upon exposure to light, respectively. Uncaging of the binding sites is mediated by a conformational change (unhinging) in the Jα helix of *As*LOV2, causing the helix to bend outward and thereby releasing the fusion domain (Figure 3b).

There is a whole set of light‐inducible systems that follow the general principle of light‐triggered heterodimerization. Depending on the desired wavelength and type of application, one can choose from a long list, including (but not limited to) UVR8–COP1, CRY2–CIB1, PhyB–phytochrome interacting factor 6 (PIF6), and BphP1–PpsR2 (Figure 2a). Since UVR8–COP1 is exclusively activated by UV light, it can be easily used in combination with other photoactivatable systems (Figure 2b). Also, the PhyB–PIF6 system associates and dissociates upon exposure to red and far‐red light, respectively. In other words, red light (≈650 nm) converts the inactive (Pr) state of the PhyB into the active (Pfr) state by *cis*–*trans* isomerization of the cofactor PCB, whereas far‐red light (750 nm) promotes the reverse transition. Interestingly, both association and dissociation can occur on a timescale of milliseconds, which makes it possible to precisely regulate protein–protein interaction.^87^ A drawback of this system is the need to externally provide the PCB chromophore in mammalian cells. If optical induction of transgene expression in vivo and in deep tissue is intended, BphP1–PpsR2 promises to be a reliable system due to its near‐infrared absorption spectrum (light in this region shows high tissue penetration)^71^ (Figure 2b).

In contrast to heterodimerization, the light‐dependent formation of homodimers is a common strategy for light sensing in nature. The underlying mechanisms and kinetics of dimerization have been intensively studied, as exemplified by the VVD domains of the *Cr*LOV protein from *Neurospora crassa*,^49^, ^88^ as well as *Rs*LOV from *Rhodobacter sphaeroides*.^46^ Coupling of such proteins with unrelated signaling pathways enables the design of novel signaling entities. Fusion of the intracellular domain of FGFR1 to the LOV domain of aureochrom 1 (AU1), for example, enables reversible dimerization and activation of the receptor by blue light^63^ (Figure 3b).

Some photoactivatable proteins, such as CRY2, naturally homo‐oligomerize in large clusters upon light activation. This behavior can be used, for instance, in systems where the activity of the effector protein relies on close proximity, or a high effector domain concentration, for reciprocal activation. Thus, fusion of the effector protein to CRY2 can result in a light‐inducible switch that activates signal transduction^67^ (Figure 3a).

Protein sequestration is another strategy that can be used to remove proteins from their site of action (in order to block their activity), and could become a platform for photoactivation of proteins of interest by light‐induced dissociation.^61^ Dronpa145N,^73^ *Rs*LOV,^78^, ^82^ UVR8,^89^ and ZdK/*As*LOV2^90^ have been used as dissociation switches for different applications (Figure 3a).

## 5 Optogenetic Applications

### 5.1 Light‐Induced Gene Regulation and Protein Function

Photoactivated gene expression can be achieved by light‐induced homo‐/heterodimerization or recruitment of transcription factors to their respective promoter.^32^, ^40^

Flavin‐binding Klech‐repeat F‐box 1 (FKF1) from *A. thaliana* was the first photoactivatable system to be used for driving gene expression in mammalian cells.^91^ In its native environment, FKF1 has a LOV domain bearing a FMN chromophore and forms a heterodimer with the unique plant‐specific nuclear protein GIGANTEA (GI) upon activation. Yazawa et al. showed that a fusion of GI and the bacterial DNA‐binding domain of Gal4 can bind to the Gal4‐responsive upstream activating sequences (UAS) promoter element.^91^ Accordingly, interaction of orthogonally expressed GI–Gal4 and FKF1–VP16 (a transactivator domain) activates gene transcription from the UAS promoter in mammalian cells upon exposure to blue light. Similar systems have also been constructed using PhyB–PIF6, CRY2–CIB1, LOV domain (VVD and the light‐activated DNA‐binding protein EL222), and PhyB–PIB6.^32^, ^40^ As already mentioned, membrane‐bound photoreceptors can also be engineered to harness different wavelengths and pathways in order to trigger gene expression^21^ (Figure 3b).

The tunable light‐controlled interacting protein tag (TULIP) system is a versatile and tunable optogenetic tool for protein localization to specific regions, and can be used to trigger specific cellular signaling pathways. TULIPs are based on interaction between *As*LOV2 and engineered PDZ (ePDZ) domains.^92^ Here, a peptide epitope is caged by *As*LOV2 and upon blue‐light activation, Jα of the *As*LOV2 unlocks and exposes the peptide for binding by ePDZ. Thus, targeting *As*LOV2 to different regions (e.g., plasma membrane) and fusion of ePDZ to an effector protein can recruit this protein to a specific region within the cell.

Besides its utility for light‐induced gene expression to study cellular functions, optogenetics can also be used to drive the production of pharmaceutical recombinant proteins. For example, researchers have functionally rewired a blue light–activated melanopsin receptor to drive transgene expression in different mammalian cells,^21^ and these engineered cells are used for light‐controlled production of biopharmaceutical products in bioreactors. This is of particular interest to the pharmaceutical industry, because this method can help to reduce the need for sophisticated and expensive downstream processing to remove impurities derived from chemical inducers. Melanopsin‐engineered cells have also been used for therapeutic purposes, as will be discussed in detail below.

In addition to the above mechanisms, optogenetics is also able to control protein functions through modulation of protein trafficking, protein degradation, and protein clustering^26^, ^40^ (Figure 3a). For example, Chen et al. have developed a light‐controlled secretion system by fusing tandems of UVR8 to vesicular stomatitis virus glycoprotein (VSV‐G).^89^ In the dark, the UVR8 chains form oligomers which trap the fusion constructs in the ER. After stimulation with light, oligomers are dissociated and move to the Golgi apparatus and the plasma membrane. Also, a light‐regulated protein degradation system has been developed by Bonger et al.^93^ They fused a degradation signal sequence (RRRG) C‐terminally to the *As*LOV2 domain. This protein tag was named blue‐light–inducible degradation (B‐LID), and works via exposure of the degradation signal sequence in response to blue light; this leads to degradation of the tagged protein (Figure 3a).

### 5.2 Light‐Induced Cell‐Signaling Engineering

Optogenetic tools can be used to specifically modulate intracellular signaling pathways in engineered mammalian cells in a precise location at a given time, which greatly reduces off‐target effects. Indeed, light‐controlled activation of a variety of signaling pathways in mammalian cells has been successfully achieved, including, but not limited to, nuclear factor of activated T cells (NFAT), mitogen‐activated protein kinase 1 (MAPK), phosphoinositide 3‐kinase (PI3K), Ras homolog gene family, member A (RhoA), Rac1, cAMP response element (CRE), and the apoptotic caspase pathway; some of these will be discussed below.^26^

Membrane recruitment of key players and subsequent activation of the MAPK and PI3K pathways based on PhyB–PIF6 are good examples. To this end, PhyB was anchored to the plasma membrane and PIF6 was fused to either son of sevenless (SOS) protein (a MAPK pathway activator)^94^ or p85α (a PI3K‐binding protein).^95^ In these studies, PhyB–PIF6 interaction and subsequent recruitment of effector proteins to the plasma membrane were induced by red light. The PhyB–PIF6 system has also been established for light‐induced regulation of cell mobility by fusing PIF6 to three members of the Rho GTPase family; Rac, cdc42, and Rho. Rac and Cdc42 plasma membrane recruitment led to lamellipodia and filopodia formation, while Rho recruitment caused cell‐body contraction.^96^ More recently, PhyB–PIF6 interaction has also been used for efficient light‐triggered viral gene delivery. In this case, adeno‐associated viruses (AAVs) were engineered to express PIF6 in their capsid. Interaction between the PIF6 moieties in the virus capsids and PhyB (equipped with a NLS) residing in the host cell facilitated translocation of viruses to the nucleus of transduced cells.^97^

The CRY2 domain shows prominent homo‐oligomerization behavior upon exposure to blue light, as well as heterodimerization with CIB1. Good examples of homo‐oligomerization‐based clustering effects can be found in studies performed by Bugaj et al., and include photoactivation of RhoA and the β‐catenin pathway, as well as activation of the membrane receptors FGFR1 and platelet‐derived growth factor receptor (PDGFR).^67^, ^98^

Light‐induced clustering of photoativatable proteins can also be used to achieve gain‐ or loss‐of‐function. Engineered light‐inducible gain‐of‐function is illustrated by the blue light–triggered association of CRY2 to modulate the activity of β‐catenin to regulate transcription in mammalian cells.^67^ Alternatively, clustering can be used to sequester proteins from their site of action. For example, the light‐activated reversible inhibition by assembled trap (LARIAT) system has been developed to inhibit proteins that modulate cytoskeleton components, lipid signaling, and the cell cycle. The LARIAT system consists of two modules: a multimeric protein (p) and a light‐mediated heterodimerizer. Here, Ca^2+^‐/calmodulin‐dependent protein kinase IIα (CaMKIIα) as the multimeric protein is fused to CIB1. The heterodimerizing protein CRY2 is coexpressed as a binding partner for CIB1. In the dark state, CaMKIIα as the multimeric p is active, but upon stimulation with blue light, CRY2 binds to the fusion protein and induces oligomerization into large clusters, thereby inhibiting CaMKIIα.^99^

Systems based on uncaging can also be used for light‐induced cell signaling in mammalian cells. A good example of light‐induced uncaging to control cellular behavior is the fusion of *As*LOV2 and the small GTPase Rac1 in order to build a photoactivatable Rac1. In the dark, binding of Rac1 to its effector is blocked by the interaction of the LOV domain with Rac1. However, blue light releases this inhibition and exposes Rac1 to the effector. This approach was used for local remodeling of the cytoskeleton and for triggering cellular movement toward the illuminated region.^84^ (Figure 3a). Another possible output in this context is programed cell death of mammalian cells with temporal and spatial selectivity. It has been shown that fusion of LOV2 to caspase‐7^100^ and caspase‐3^101^ can induce cell death in targeted cells both in vitro and in vivo in a blue light–dependent manner.

Calcium acts as a second messenger to regulate a plethora of cellular activities.^100^ Three photoactivatable tools to control calcium signaling in mammalian cells are genetically encoded photoactivatable Ca^2+^ releaser (PACR),^102^ OptoSTIM1,^103^ and OptoCRAC.^104^, ^105^ PACR was developed by Fukuda et al. by inserting a LOV2 domain into the calmodulin–M13 fusion protein. In the dark, the calmodulin–M13 complex chelates Ca^2+^ with high affinity. This complex is destroyed upon illumination, which induces conformational changes in the LOV2 domain, resulting in Ca^2+^ release from the calmodulin moiety and causing a transient increase of Ca^2+^ concentration.^102^

Combinations of CRY2PHR with the stromal interaction molecule (STIM1) and with a LOV2–SOAR (the STIM1‐Orai activating region) fusion protein have both been used to modulate the activity of Ca^2+^ release–activated Ca^2+^ (CRAC) channels, as employed in the OptoSTIM1^103^ and OptoCRAC systems,^104^, ^105^ respectively. OptoSTIM1 and OptoCRAC are both calcium channels that can ultimately trigger Ca^2+^ influx via photostimulation.^106^

### 5.3 Light‐Activated Genome Engineering

**Figure 4:**
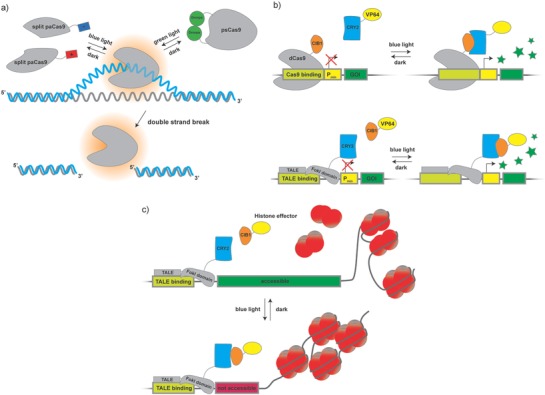
Photoregulatable Cas9 and TALE: the versatile genome‐editing system CRISPR/Cas9 provides highly precise binding to specific sequences in genomic or plasmid DNA. a) Recently, the catalytic moiety of the system, the SpCas9 enzyme, was engineered to be light sensitive by applying the heterodimerization and uncaging approach. To engineer a photoactivatable dimerizing variant of SpCas9, called paCas9, wild‐type SpCas9 was split in two parts that were fused to either pMag or nMag tags. Upon exposure to blue light, the pMag and nMag tags dimerize to reconstitute functional paCas9 that is able to bind to and cleave DNA. A different strategy was used to block the DNA‐binding site of SpCas9 via a Dronpa dimer that was fused to the wild‐type enzyme. Here, Dronpa dissociates upon stimulation with blue light, uncovering the binding domain and allowing the functional enzyme to work on the DNA. This fusion construct was termed photoswitchable Cas9 (psCas9). b) A catalytically dead version of SpCas9 (dCas9) was used to engineer a light‐inducible gene expression system based on CRY2–CIB1 interaction. In this example, CIB1 was fused to dCas9, and CRY2 was used to bind a transactivator to the DNA‐binding complex, while another system termed light‐inducible transcriptional effector (LITE) used engineered TALE proteins fused to CRY2 to bind CIB1 fused to the orthogonal transactivator VP64. While paCas9 and psCas9 are both blocked from binding to DNA, the dCas9 as well as the TALE component of these photoswitchable transcription systems resides on the DNA in the dark. Exposure to blue light recruits CRY2–VP64 to dCas9–CIB1 and CIB1–VP64 to TALE–CRY2, triggering assembly of the transcription initiation complex. c) In the epiLITE system, the aforementioned system was used to recruit inactivating epigenetic effectors (e.g., deacetylases, methylases) to a defined region on the genome, resulting in reduction of gene expression from the targeted locus.

The clustered regularly interspaced short palindromic repeat (CRISPR)/Cas9 system is a simple and powerful tool to introduce alterations in genomic DNA or its epigenetic context.^107^ This system is capable of silencing, enhancing, removing, adding, or modulating genes.^107^, ^108^ The CRISPR/Cas9 system is an effective tool to understand complex gene networks, and also has great potential in medical and industrial applications^109^ (**Figure** **4**).

A combination of CRISPR with optogenetics uses light to provide external control of the location and timing of genome editing, as well as modulating Cas9‐mediated transcriptional activity. Nihongaki et al. developed a photoactivatable Cas9 (called paCas9) for light‐induced gene deletion and insertion.^110^ In this system, Cas9 was split into N‐terminal and C‐terminal halves and each part was fused to nMag or pMag of the Magnet system^79^ mentioned previously in this Review. Blue light induces heterodimerization between pMag and nMag, which leads to the assembly of a functional SpCas9 protein that cleaves DNA at a user‐defined place in the genome (Figure 4a).

The fusion of wild‐type SpCas9 to dimerized pdDronpa domains provided a single‐chain photoswitchable Cas9 (psCas9), which harnesses the principle of uncaging.^111^ In the dark, pdDronpa domains form a dimer that efficiently prevents binding of SpCas9 to DNA. Upon exposure to green light the dimer dissociates, re‐establishing a functional SpCas9 complex that is able to induce double‐strand breaks in DNA. The same system can be adapted to enable light‐induced gene expression. To this end, a catalytically dead version of SpCas9 (dCas9), that still retains its DNA‐binding properties, was equipped with Dronpa‐based blocking domains and fused to the transcriptional activator viral protein R (VPR). As before, dCas9 is not able to bind to DNA in the dark, but regains full activity upon cyan light–induced dissociation of the Dronpa dimer (Figure 4a). In another approach, a combination of dCas9 with light‐controlled CRY2–CIB1 recruitment enables activation of target genes from their endogenous loci. To achieve this, Nihongaki et al. fused dCas9 to CIB1 (dCas9–CIB1) and CRY2 to either of the transactivators VP64 or p65 (CRY2–VP64/CRY2–p65). Illumination of mammalian cells transfected with both constructs led to significantly enhanced expression of the targeted *achaete‐scute homolog 1* (*Ascl1*) gene^112^ (Figure 4b).

Similarly, Polstein and Gersbach have developed a light‐activated CRISPR/Cas9 effector (LACE) system.^113^ In their system the N‐terminal fragment of CIB1 was fused to both ends of dCas9 to form CIBN–dCas9–CIBN. The binding partner of CIB1, CRY2, was fused to the transactivator VP64 (CRY2–VP64). This system was designed to target the *interleukin 1 receptor antagonist* (*IL1RN*) gene and was able to increase its expression significantly upon illumination.

Light‐dependent activation of transcription of endogenous genes through the alternative nuclease system transcription activator–like effector nuclease (TALEN) and CRY2–CIB1 complement the available toolbox.^82^ Here, light‐induced interaction between CRY2 and CIB1 fused to TALENs was harnessed to drive transcription from the endogenous locus of an important mediator of neuronal fate, Neurogenin‐2 (Neurog2), in primary mouse neurons (Figure 4b).

The precise manipulation of chromatin and epigenetic modifications is an important goal in epigenetic engineering. Light‐induced temporal control of such modifications can provide information about the stability and kinetics of epigenetic systems.^114^ In order to optically control mammalian endogenous transcription and epigenetic states, an optogenetic two‐hybrid system based on customizable TALE DNA‐binding domains and the CRY2–CIB1 system, named light‐inducible transcriptional effectors (LITEs), was developed.^82^ Fusions of TALE to a CRY2PHR domain and of CIB1 to either VP64 or a histone effector have been used for modulation of endogenous transcription or epigenetic modifications (e.g., repression) in the desired loci in the genome of mammalian cells (Figure 4c).

### 5.4 Light‐Engineered Cells in Drug Discovery and Translational Medicine

Drug screening can be both tedious and costly, but optogenetics provide a cheap, simple, and noninvasive way to facilitate the process.^115^ As an example, Inglés‐Prieto et al. have recently developed a light‐assisted cell‐based screening system for small molecules; this enabled identification of inhibitors that interfere with pharmaceutically relevant pathways of different receptor tyrosine kinases (RTKs) through a synthetic biological circuit.^116^ They prepared three light‐sensitive Opto‐RTK receptors (Opto‐mFGFR1, Opto‐hEGFR (human epidermal growth factor receptor), and Opto‐hROS1 (proto‐oncogene tyrosine–protein kinase reactive oxygen species (ROS))), which are inert to their natural ligands, but are stimulated by light (Figure 3b). The intracellular parts of these receptors are fused to the LOV domain of aureochrome 1 from *Vaucheria frigida*, and remain inactive in the dark. Upon illumination, the inhibitory effect of the LOV domain is removed, leading to receptor dimerization and subsequent activation of the mitogen‐activated protein (MAP) kinase signaling pathway. This pathway was rewired to express a *gfp* gene from a serum response element (SRE) promoter. In this context, RTK pathway inhibitors can inhibit expression of green fluorescent protein (GFP), allowing identification of potential inhibitor candidates. Interestingly, since optogenetics‐based drug screening relies solely on light induction and there is no need for the natural ligand to induce the downstream pathway, it can even be used in drug screening for orphan receptors, whose ligands are unknown.

In a clinical setting, both direct and indirect applications of optogenetics can be envisioned.^31^, ^117^ In the direct approach, cells inside the body need to be transduced with a vector encoding a light‐sensitive protein. These cells can then be stimulated and controlled via an external device (e.g., goggles that deliver light to the eyes).^118^ In an indirect approach, optogenetics would be combined with cell‐based therapy, where cells are made sensitive to light ex vivo and can be transplanted back into the body after quality control assessment. One of the best candidates for optogenetics therapy is retinitis pigmentosa (RP), an inherited retinal degeneration disorder. Currently, a clinical trial is underway based on delivery of optogenetic channels (e.g., channelrhodopsin) to retinal cells in order to resensitize these cells to light.^117^, ^118^

Light‐sensitive engineered cells for cell therapy can be divided into two groups: primary host‐derived cells (e.g., T cells or stem cells) and engineered designer cells originating from previously established cell lines. Design of photoactivatable host‐derived cells such as T cells, called opto‐immunoengineering,^119^ can enable selective activation of these cells only at the intended location (e.g., a tumor), thus reducing off‐target effects. For example, the photoactivatable chemokine receptor 4 (PA‐CXCR4) is able to optogenetically control migration of T cells toward cancer cells both in vitro and in vivo upon illumination.^120^

**Figure 5:**
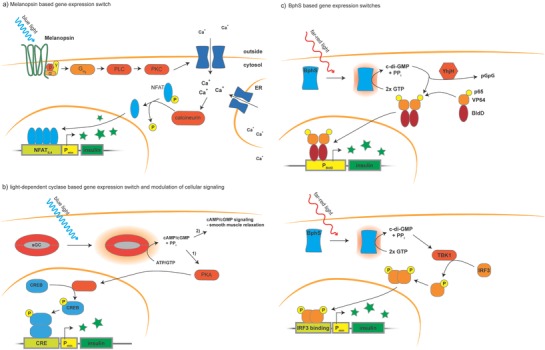
Optotherapeutic strategies: several optogenetic implants have already been tested in mice and proved to be functional to treat diabetes as well as erectile dysfunction. a) An implant harboring synthetic mammalian designer cells equipped with blue light–induced melanopsin signals through the protein kinase C (PKC) signaling pathway. Activation of melanopsin results in influx of Ca^2+^ ions from outside of the cells and release of calcium from the ER. Ultimately, this leads to activation of the phosphatase calcineurin, which dephosphorylates NFAT and triggers expression of insulin from a synthetic NFAT‐responsive promoter. b) To tackle the delicate issue of erectile dysfunction, a blue light–inducible soluble guanylate cyclase (sGC) of bacterial origin was engineered to favor GTP over ATP as a substrate. 1) To evaluate the performance of the system more easily in cell culture, cAMP levels were read out by the cAMP‐responsive CRE pathway. 2) Activation of the enzyme also directly leads to increased cGMP levels, triggering relaxation of smooth muscle cells. To avoid side effects, this system was not used as a cell‐based therapy, but was integrated directly into smooth muscle cells in the endothelium of the corpora cavernosum of rats. c) Two other approaches capitalized on the soluble c‐di‐GMP synthase BphS, which can be directly activated by far‐red light. In one system, c‐di‐GMP activated a chimeric transcription factor based on the bacterial protein BldD that dimerizes and binds to the DNA in a c‐di‐GMP‐dependent manner and is fused to the transactivators VP64 and p65. To avoid overproduction of c‐di‐GMP and overactivation of the system, YhjH was coexpressed; it hydrolyzes c‐di‐GMP to form inactive pGpG. In another study, c‐di‐GMP was used as an inducer of the endogenous STING pathway, which activates the transcription factor interferon regulatory factor 3 (IRF3) through phosphorylation by TANK‐inding kinase 1 (TBK1). Activated IRF3 binds to IRF3‐binding sites on a synthetic promoter and triggers insulin expression.

However, engineering of autologous cells is both tedious and inefficient. Instead, engineering‐immortalized mammalian cells that have been developed to behave in a predictable way is more convenient.^121^ Since such designer cells can be recognized and attacked by the host immune system, encapsulation of these cells into semipermeable polymers is required prior to transplantation into the body.^31^ Pioneering work on optogenetically engineered designer cells to maintain blood glucose homeostasis in type 2 diabetic (T2D) mice was done by Ye et al.^21^ (**Figure** **5**a). They constructed a photosensitive mammalian designer cell system by heterologous expression of melanopsin. The expression of melanopsin generates a blue light–dependent calcium influx into the cells. This feature enabled the cells to rewire the signal transduction of light‐activated melanopsin to NFAT in order to trigger expression of glucagon‐like peptide 1 (GLP‐1). Following in vitro assessment, these engineered designer cells were encapsulated and implanted into T2D mice. Under transdermal illumination, the diabetic mice showed a reduction of blood glucose due to the expression of GLP‐1.

Using the same spectrum of light for stimulation, Kim et al. also developed an optigenetic‐based erectile stimulator that can trigger an erection in male rats^122^ (Figure 5b). This system was called erectile optogenetic stimulator (EROS) and is based on an optimized light‐inducible guanylate cyclase, which produces cGMP from GTP upon exposure to blue light. cGMP, in turn, causes a reduction of the interacellular calcium concentration by closing calcium channels in the plasma membrane. This synthetic genetic surrogate thereby ultimately activates the natural process of vasodilation via the nitric oxide (NO) pathway, leading to penile erection in vivo. Light‐activated cGMP levels were monitored by a chimeric promoter based on the glutathione‐S‐transferase alpha 4 (GTA4) promoter that drives the expression of the secreted reporter protein secreted embryonic alkaline phosphatase (SEAP) in a cGMP‐dependent manner for initial assessement of the implant functionality.

Another light‐induced transgene expression system that was developed to modulate therapeutic gene expression is the LightOn system.^62^ Wang et al. constructed a photoactivatable gene switch composed of Gal4(65), the VVD domain and the p65 or VP16 activation domain. The DNA‐binding domain comprising Gal4 residues 1–147, Gal4(147), consists of a DNA‐recognition element and a dimerization domain. Further removal of the dimerization domain yielded Gal4(65) with only residues 1–65 remaining. Interestingly, this construct was no longer able to bind its consensus cognate DNA sequence, the upstream activating sequence of Gal (UASG). However, fusion of VVD domains to Gal4(65) and the transactivators p65 or VP16 yielded the construct Gal4(65)–VVD–p65/VP16 (GAVP or GAVV, respectively), which regained its DNA‐binding ability in a blue light–dependent manner. Upon exposure to blue light, GAVP or GAVV homodimerizes, and the homodimer interacts with UASG elements (5× UASG) to initiate expression of the gene of interest. This system successfully reduced blood glucose levels in diabetic mice via blue light–controlled insulin expression.

Critically, however, these blue light–induced systems suffer from the inherent low penetration of blue light into tissues. This results in higher cytotoxicity, since increased doses of irradiation are necessary to reach the cells.

More recently, Shao et al. succeeded in developing a far‐red light–inducible expression system, which affords reduced cytotoxicity and offers improved tissue penetration^19^ (Figure 5c, top). In addition, they developed a smartphone‐controlled optogenetic designer cell device that can semiautomatically monitor and control blood glucose homeostasis in diabetic mice. In this system, designer cells contain bacterial phytochrome cyclic‐di‐guanosine monophosphate (c‐di‐GMP) synthase (BphS), which produces c‐di‐GMP from GTP upon exposure to far‐red light. c‐di‐GMP binds to a c‐di‐GMP‐binding transcription factor, called BldD, which is fused to a transactivator domain (p65–VP64). Upon activation, BphS converts GTP into c‐di‐GMP, which induces the formation of BldD–p65–VP64 dimer, which can bind to a synthetic promoter. This system has been successfully used for regulation of gene transcription (e.g., to express GLP‐1 or insulin). They also tested the feasibility of controlling intracellular c‐di‐GMP levels via YhjH (PdeH), which is a bacterial c‐di‐GMP phosphodiesterase that converts c‐di‐GMP to P‐(5′‐guanosyl)‐P‐(5′‐guanosyl)‐(3′‐5′)‐diphosphate (pGpG) and contributes to the regulation of *Escherichia coli* motility.^82^ This BphS and BldD–p65–VP64‐dependent genetic circuit has been implemented in mammalian designer cells, where it was controlled with a custom‐engineered Bluetooth‐compatible glucometer in a semiautomatic manner: the glucometer monitors blood glucose levels in the body and sends the data to a SmartController or a smartphone linked via Bluetooth. The SmartController translates the input into corresponding far‐red light signals of appropriate intensity, which can be sensed by insulin‐producing implanted designer cells to mediate blood glucose homeostasis.^19^, ^123^

A major step toward the application of optogenetics for next‐generation synthetic biology‐inspired medicine is mind‐controlled gene expression, which was successfully implemented in mice by Folcher et al.^60^ They developed light‐controlled designer cells by introducing light‐activated bacterial BphG1 DGCL; this produces c‐di‐GMP, which triggers the stimulator of interferon genes (STING)–dependent expression of SEAP from a synthetic interferon‐β promoter. This system was induced by 700 nm near‐infrared (NIR) light to express SEAP in human embryonic kidney 293 (HEK293) cells. Next, they placed these light‐responsive designer cells in a cartridge and implanted it under the skin of mice along with a NIR–light‐emitting diode (LED). To test the scope of the implant, they recorded the brain activity of trained volunteers with an electroencephalograph headset connected to a computer. Based on preset thresholds, the computer turned off or turned on an electrical‐field generator in close proximity to mice carrying the implant. This generator in turn powered up the LED within the implant and provided light to induce the photoresponsive designer cells, leading to production and subsequent release of SEAP into the bloodstream of the mice (Figure 5c, bottom).

## 6 Challenges in Optogenetic‐Based Therapeutical Mammalian Synthetic Biology and Possible Solutions

To move beyond proof‐of‐concept studies on light‐controlled mammalian designer cells, two main problems should be considered carefully: safe and efficient implantation of light‐controlled designer cells into the body, and effective light delivery for activation of photosensitive gene circuits in the implanted designer cells.

So far, most of the available synthetic gene circuits, especially those for treating metabolic diseases, have been implemented in cell lines such as HEK293 cells.^124^ The use of these cell lines requires microencapsulation to protect the cells from the host immune system. Implants consisting of engineered microencapsulated designer cells have already been used to introduce photosensitive cells with predefined functions into the host body.^31^, ^125^ Although cell microencapsulation offers well‐known advantages, e.g., protection from the host immune system, along with controlled and cost‐effective therapeutic product delivery, there are several issues that still need to be addressed, including capsule manufacturing, properties, and performance.^82^

Systems based on induced pluripotent stem cells (iPSC) or even primary cells (e.g., mesenchymal stem cells) should provide a new platform for the next generation of photosensitive designer cells, thereby reducing concerns and side effects associated with implantation of immortalized cell line–based designer cells.^123^

**Figure 6:**
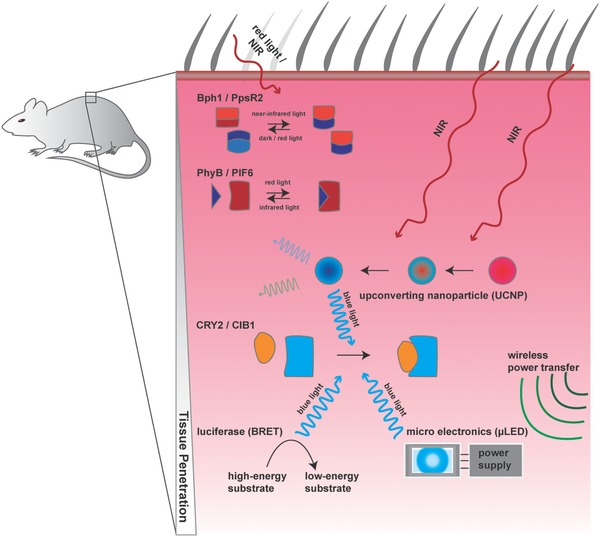
State of the art of in vivo optogenetics: applications of optogenetics in vivo have to deal with the issue of tissue penetration of light and potential phototoxicity. Many optogenetic systems are dependent on high‐energy blue and green light, which does not penetrate deeply into tissues. Constructs that rely on red and near‐infrared light, such as the Bph1/PpsR2 or PhyB/PIF6 system, can be used in deep‐layer implants, because tissue penetration of light generally increases with wavelength. However, to expand the selection of optogenetic tools available for more advanced and multiplexed circuits, several solutions have been introduced by the scientific community. To enable activation of blue and green light–dependent circuits, upconverting nanoparticles (UCNPs) can be used. These nanoparticles collect energy from more than one photon and emit a single photon with a shorter wavelength. A straightforward approach to circumvent the tissue barrier is to directly equip the implant with its own light source that can be powered wirelessly via electromagnetic induction. With the advent of very small and efficient micro LEDs (µLEDs), this strategy is becoming increasingly feasible. Luminopsin is another tool that uses chemical energy stored in small molecules to drive light‐powered reactions. This is achieved by the localization of luciferases in the vicinity of the photoactivatable protein. Luciferases convert the exogenously provided high‐energy substrate coelenterazine into the low‐energy product coelenteramide while simultaneously transferring the light‐energy produced by the reaction to a nearby chromophore in a photosensitive protein in a process called bioluminescence resonance energy transfer (BRET).

Designer cell implants are most often located in deeper layers of tissue where they can barely be reached by light from the outside. So far, four optogenetic strategies have been developed to cope with such in vivo applications (**Figure** **6**). First, photosensitive proteins can be selected that are excited by light of a longer wavelength, which can penetrate tissue more easily (since blue light has a low wavelength and a high energy, the use of blue light leads to both low penetration efficiency and higher photo‐cytotoxicity). PhyB–PIF and BphP1–PpsR2 are two systems which provide excellent tissue penetration in the red and near‐infrared ranges. Second, lanthanide‐doped nanoparticles have the ability to absorb low‐energy near‐infrared light and emit higher‐energy blue or green light in a process called upconversion.^126^ The introduction of upconverting nanoparticles (UCNPs) in the field of optogenetics made it possible to indirectly activate blue/green light–absorbing photosensitive proteins even in deep layers of tissue.^127^ Upconverting nanoparticles paired with the Opto‐CRAC system have been used to control Ca^2+^ signaling in immune cells in vivo.^105^ Third, in a more straightforward approach, implementation of micro LED (µLED) implants that can be controlled and powered by radio frequencies can provide light in deep layers of tissue.^128^, ^129^, ^130^ Finally, bioluminescence from luciferases can also be used as an alternative light source for photoactivation of blue/green light–sensitive proteins.^131^ Here, a luciferase is fused to microbial rhodopsin, giving rise to luminopsin. The luciferase moiety of luminopsin catalyzes the oxidation of its substrate coelenterazine to coelenteramide. This process generates light as a by‐product which is further transferred via a Förster‐resonance electron transport (FRET)–like mechanism to the attached microbial rhodopsin.^83^, ^119^

## 7 Conclusions and Future Directions

The field of optogenetics emerged from pioneering work using light‐gated ion channels to manipulate the activity of neural cells.^132^ Subsequently, collaborations between neurobiologists and other cell biologists have adapted optogenetics to other cell types and tissues, making it possible not only to change the action potential across plasma membranes, but also to drive therapeutic gene expression and many other cellular functions. Compared to chemically controlled systems or conventional genetic engineering methods, optogenetics provides far more flexible and precise spatial and temporal control of signaling dynamics, protein levels, and enzyme activity, with minimal side effects.^32^, ^40^ On the other hand, synthetic biology‐inspired therapeutic strategies with engineered designer cells have provided new opportunities for treating diseases, such as metabolic disorders,^21^ cancer,^15^, ^133^ and blindness.^75^ Thus, current work aims to employ the combination of optogenetics with designer cells to achieve precise control of therapeutic outputs by light (blue light,^21^, ^62^ red light,^134^ infrared light,^60^ or far‐red light^135^).^123^

The rapidly growing number of available optogenetic tools offers an opportunity to synthetic biologists to choose the best approach and strategy for their particular purposes. The wavelength of activating light, the endogenous availability of chromophores, and the reversibility and dynamics of the optogenetic system are some of the most important parameters that should be taken into account during the selection of optogenetic devices.^32^

Cell‐based therapy is considered a very promising approach for next‐generation medicine.^136^ Some designer cells such as Kymriah, a preparation of T cells expressing a chimeric antigen receptor (CAR), are already on the market. However, a non‐negligible side effect associated with CAR‐expressing cells is unspecific cytotoxicity (“on‐target‐off‐tumor” toxicity). It should be possible to apply optogenetics to improve the safety of CAR technology by enabling user‐defined light‐controlled activation of designer T cells exclusively at the site of the tumor.^119^ Near‐infrared‐light‐inducible BphP1–PpsR2 is emerging as a promising tool that is consistent with deep tissue penetration of the light stimulus, and should be especially useful for therapeutic synthetic biology.

**Figure 7:**
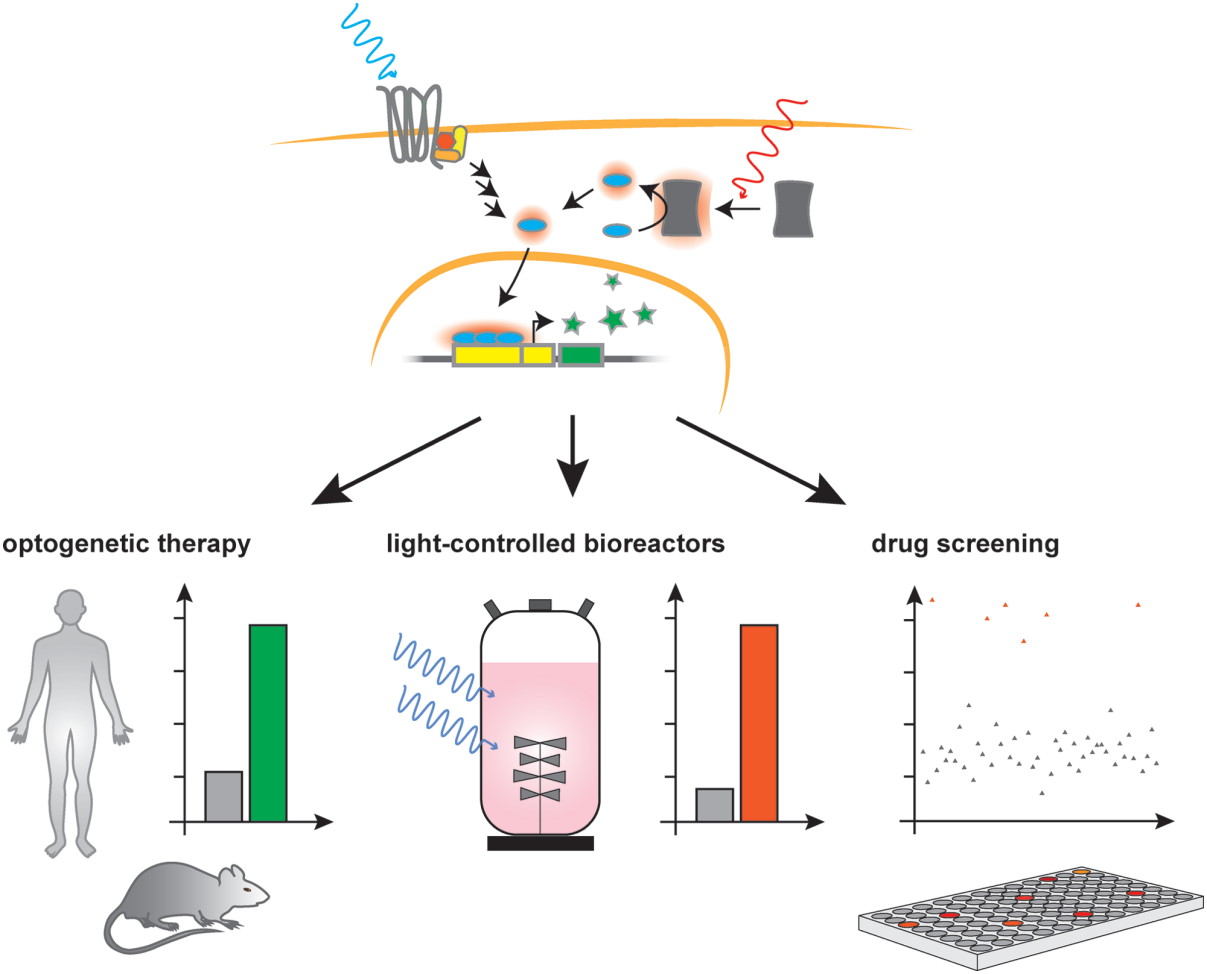
Optogenetics in translational mammalian synthetic biology: the use of light‐controlled mammalian designer cells is gaining broad acceptance, with numerous applications appearing every year. Optogenetic therapy can lead to precise and specific treatment with reduced side effects in humans and in animal models. Additionally, light‐controllable production of pharmaceuticals reduces the need for external inducers that might cause problems in downstream processing. The use of light‐controlled signaling pathways for the implementation of ligand‐free, cost‐effective, and efficient screening systems can promote drug development.

Moving beyond cell‐based therapy, commercial production of biopharmaceuticals in a light‐inducible bioreactor without any chemical inducer can facilitate downstream processing and reduce the number of purification steps needed in the manufacture of bioproducts (**Figure** **7**).

Finally, we believe that the combination of optogenetics and synthetic biology will play a major role in opening up new applications, not only in the clinic and pharmaceutical industry, but also in basic research.

## Conflict of Interest

The authors declare no conflict of interest.

## References

[1] M. Gross , Light and Life, Oxford University Press, Oxford 2002.

[2] M. F. Hohmann‐Marriott , R. E. Blankenship , Annu. Rev. Plant Biol. 2011, 62, 515. 21438681 10.1146/annurev-arplant-042110-103811

[3] Y. Fu , in Webvision: The Organization of the Retina and Visual System (Eds: Kolb H., Fernandez E., Nelson R.), University Of Utah Health Sciences Center, UT 1995. 21413389

[4] H. G. McWatters , P. F. Devlin , FEBS Lett. 2011, 585, 1474. 21453701 10.1016/j.febslet.2011.03.051

[5] R. S. McIsaac , C. N. Bedbrook , F. H. Arnold , Curr. Opin. Struct. Biol. 2015, 33, 8. 26038227 10.1016/j.sbi.2015.05.001 PMC4641784

[6] F. Zhang , J. Vierock , O. Yizhar , L. E. Fenno , S. Tsunoda , A. Kianianmomeni , M. Prigge , A. Berndt , J. Cushman , J. Polle , J. Magnuson , P. Hegemann , K. Deisseroth , Cell 2011, 147, 1446. 22196724 10.1016/j.cell.2011.12.004 PMC4166436

[7] M. Banghart , K. Borges , E. Isacoff , D. Trauner , R. H. Kramer , Nat. Neurosci. 2004, 7, 1381. 15558062 10.1038/nn1356 PMC1447674

[8] K. Deisseroth , Sci. Am. 2010, 303, 48. 10.1038/scientificamerican1110-48 21033283

[9] E. S. Boyden , F. Zhang , E. Bamberg , G. Nagel , K. Deisseroth , Nat. Neurosci. 2005, 8, 1263. 16116447 10.1038/nn1525

[10] K. Deisseroth , G. Feng , A. K. Majewska , G. Miesenbock , A. Ting , M. J. Schnitzer , J. Neurosci. 2006, 26, 10380. 17035522 10.1523/JNEUROSCI.3863-06.2006 PMC2820367

[11] L. Fenno , O. Yizhar , K. Deisseroth , Annu. Rev. Neurosci. 2011, 34, 389. 21692661 10.1146/annurev-neuro-061010-113817 PMC6699620

[12] A. S. Khalil , J. J. Collins , Nat. Rev. Genet. 2010, 11, 367. 20395970 10.1038/nrg2775 PMC2896386

[13] S. Ausländer , D. Ausländer , M. Fussenegger , Angew. Chem., Int. Ed. 2017, 56, 6396. 10.1002/anie.201609229 27943572

[14] W. Weber , M. Fussenegger , Nat. Rev. Genet. 2012, 13, 21. 10.1038/nrg3094 PMC7097403 22124480

[15] K. T. Roybal , J. Z. Williams , L. Morsut , J. Rupp , I. Kolinko , J. H. Choe , W. J. Walker , K. A. Mcnally , W. A. Lim , Cell 2016, 167, 419. 27693353 10.1016/j.cell.2016.09.011 PMC5072533

[16] C. Kemmer , M. Gitzinger , M. Daoud‐El Baba , V. Djonov , J. Stelling , M. Fussenegger , Nat. Biotechnol. 2010, 28, 355. 20351688 10.1038/nbt.1617

[17] K. Rössger , G. Charpin‐El Hamri , M. Fussenegger , Proc. Natl. Acad. Sci. USA 2013, 110, 18150. 24127594 10.1073/pnas.1312414110 PMC3831458

[18] L. Schukur , B. Geering , G. Charpin‐El Hamri , M. Fussenegger , Sci. Transl. Med. 2015, 7, 318ra201. 10.1126/scitranslmed.aac4964 26676608

[19] J. Shao , S. Xue , G. Yu , Y. Yu , X. Yang , Y. Bai , S. Zhu , L. Yang , J. Yin , Y. Wang , S. Liao , S. Guo , M. Xie , M. Fussenegger , H. Ye , Sci. Transl. Med. 2017, 9, eaal2298. 28446682 10.1126/scitranslmed.aal2298

[20] M. Xie , H. Ye , H. Wang , G. Charpin‐El Hamri , C. Lormeau , P. Saxena , J. Stelling , M. Fussenegger , Science 2016, 354, 1296. 27940875 10.1126/science.aaf4006

[21] H. Ye , M. D.‐E. Baba , R.‐W. Peng , M. Fussenegger , Science 2011, 332, 1565. 21700876 10.1126/science.1203535

[22] H. Ye , M. Xie , S. Xue , G. C.‐E. Hamri , J. Yin , H. Zulewski , M. Fussenegger , Nat. Biomed. Eng. 2016, 1, 0005. 28480128 10.1038/s41551-016-0005 PMC5412959

[23] T. Kitada , B. DiAndreth , B. Teague , R. Weiss , Science 2018, 359, eaad1067. 29439214 10.1126/science.aad1067 PMC7643872

[24] H. Ye , M. Fussenegger , FEBS Lett. 2014, 588, 2537. 24844435 10.1016/j.febslet.2014.05.003

[25] A. P. Teixeira , M. Fussenegger , Curr. Opin. Biotechnol. 2017, 47, 59. 28662442 10.1016/j.copbio.2017.06.004

[26] K. Zhang , B. Cui , Trends Biotechnol. 2015, 33, 92. 25529484 10.1016/j.tibtech.2014.11.007 PMC4308517

[27] L. Scheller , T. Strittmatter , D. Fuchs , D. Bojar , M. Fussenegger , Nat. Chem. Biol. 2018, 14, 723. 29686358 10.1038/s41589-018-0046-z

[28] W. Weber , R. R. Marty , N. Link , M. Ehrbar , B. Keller , C. C. Weber , A. H. Zisch , C. Heinzen , V. Djonov , M. Fussenegger , Nucleic Acids Res. 2003, 31, 69e. 10.1093/nar/gng069 PMC162344 12799458

[29] D. Ausländer , S. Ausländer , G. Charpin‐El Hamri , F. Sedlmayer , M. Müller , O. Frey , A. Hierlemann , J. Stelling , M. Fussenegger , Mol. Cell 2014, 55, 397. 25018017 10.1016/j.molcel.2014.06.007

[30] S. A. Stanley , J. E. Gagner , S. Damanpour , M. Yoshida , J. S. Dordick , J. M. Friedman , Science 2012, 336, 604. 22556257 10.1126/science.1216753 PMC3646550

[31] D. Ausländer , M. Fussenegger , Gastroenterology 2012, 143, 301. 22721803 10.1053/j.gastro.2012.06.019

[32] K. Müller , S. Naumann , W. Weber , M. D. Zurbriggen , Biol. Chem. 2015, 396, 145. 25153239 10.1515/hsz-2014-0199

[33] G. Aston‐Jones , K. Deisseroth , Brain Res. 2013, 1511, 1. 23422677 10.1016/j.brainres.2013.01.026 PMC3663045

[34] B. V. Zemelman , G. A. Lee , M. Ng , G. Miesenböck , Neuron 2002, 33, 15. 11779476 10.1016/s0896-6273(01)00574-8

[35] G. Nagel , T. Szellas , W. Huhn , S. Kateriya , N. Adeishvili , P. Berthold , D. Ollig , P. Hegemann , E. Bamberg , Proc. Natl. Acad. Sci. USA 2003, 100, 13940. 14615590 10.1073/pnas.1936192100 PMC283525

[36] W. Bacchus , M. Fussenegger , Curr. Opin. Biotechnol. 2012, 23, 695. 22204821 10.1016/j.copbio.2011.12.004

[37] N. A. Repina , A. Rosenbloom , A. Mukherjee , D. V Schaffer , R. S. Kane , Annu. Rev. Chem. Biomol. Eng. 2017, 8, 13. 28592174 10.1146/annurev-chembioeng-060816-101254 PMC5747958

[38] A. Guru , R. J. Post , Y.‐Y. Ho , M. R. Warden , Int. J. Neuropsychopharmacol. 2015, 18, pyv079. 26209858 10.1093/ijnp/pyv079 PMC4756725

[39] A. Kaneko , K. Inoue , K. Kojima , H. Kandori , Y. Sudo , Biophys. Rev. 2017, 9, 861. 29178082 10.1007/s12551-017-0335-x PMC5711702

[40] K. Kolar , W. Weber , Curr. Opin. Biotechnol. 2017, 47, 112. 28715701 10.1016/j.copbio.2017.06.010

[41] T. Ziegler , C. H. Schumacher , A. Möglich , in Methods in Molecular Biology (Ed: Walker J. M.), Springer, Berlin 2016, pp. 389–403. 10.1007/978-1-4939-3512-3_27 26965138

[42] A. Terakita , Genome Biol. 2005, 6, 213. 15774036 10.1186/gb-2005-6-3-213 PMC1088937

[43] O. P. Ernst , D. T. Lodowski , M. Elstner , P. Hegemann , L. S. Brown , H. Kandori , Chem. Rev. 2014, 114, 126. 24364740 10.1021/cr4003769 PMC3979449

[44] S. Hattar , R. J. Lucas , N. Mrosovsky , S. Thompson , R. H. Douglas , M. W. Hankins , J. Lem , M. Biel , F. Hofmann , R. G. Foster , K.‐W. Yau , Nature 2003, 424, 75. 10.1038/nature01761 PMC2885907 12808468

[45] L. S. Mure , M. Hatori , Q. Zhu , J. Demas , I. M. Kim , S. K. Nayak , S. Panda , Neuron 2016, 90, 1016. 27181062 10.1016/j.neuron.2016.04.016 PMC4891235

[46] H. J. Bailes , L.‐Y. Zhuang , R. J. Lucas , PLoS One 2012, 7, e30774. 22292038 10.1371/journal.pone.0030774 PMC3265508

[47] K. Sasaki , T. Yamashita , K. Yoshida , K. Inoue , Y. Shichida , H. Kandori , PLoS One 2014, 9, e91323. 24621599 10.1371/journal.pone.0091323 PMC3951393

[48] K. Yoshida , T. Yamashita , K. Sasaki , K. Inoue , Y. Shichida , H. Kandori , Biophys. Physicobiol. 2017, 14, 183. 29362703 10.2142/biophysico.14.0_183 PMC5774426

[49] B. D. Zoltowski , C. Schwerdtfeger , J. Widom , J. J. Loros , A. M. Bilwes , J. C. Dunlap , B. R. Crane , Science 2007, 316, 1054. 17510367 10.1126/science.1137128 PMC3682417

[50] E. G. Govorunova , O. A. Sineshchekov , H. Li , J. L. Spudich , Annu. Rev. Biochem. 2017, 86, 845. 28301742 10.1146/annurev-biochem-101910-144233 PMC5747503

[51] K. Inoue , Y. Kato , H. Kandori , Trends Microbiol. 2015, 23, 91. 25432080 10.1016/j.tim.2014.10.009

[52] J. K. Lanyi , Annu. Rev. Physiol. 2004, 66, 665. 14977418 10.1146/annurev.physiol.66.032102.150049

[53] D. Oesterhelt , W. Stoeckenius , Proc. Natl. Acad. Sci. USA 1973, 70, 2853. 4517939 10.1073/pnas.70.10.2853 PMC427124

[54] D. Oesterhelt , W. Stoeckenius , Nature, New Biol. 1971, 233, 149. 4940442 10.1038/newbio233149a0

[55] J. M. Walter , D. Greenfield , C. Bustamante , J. Liphardt , Proc. Natl. Acad. Sci. USA 2007, 104, 2408. 17277079 10.1073/pnas.0611035104 PMC1892948

[56] K. Y. Hara , T. Wada , K. Kino , T. Asahi , N. Sawamura , Sci. Rep. 2013, 3, 1635. 23567447 10.1038/srep01635 PMC3620844

[57] V. Shevchenko , T. Mager , K. Kovalev , V. Polovinkin , A. Alekseev , J. Juettner , I. Chizhov , C. Bamann , C. Vavourakis , R. Ghai , I. Gushchin , V. Borshchevskiy , A. Rogachev , I. Melnikov , A. Popov , T. Balandin , F. Rodriguez‐Valera , D. J. Manstein , G. Bueldt , E. Bamberg , V. Gordeliy , Sci. Adv. 2017, 3, e1603187. 28948217 10.1126/sciadv.1603187 PMC5609834

[58] T. Kushibiki , S. Okawa , T. Hirasawa , M. Ishihara , Gene Ther. 2015, 22, 553. 25809465 10.1038/gt.2015.23

[59] M. Stierl , P. Stumpf , D. Udwari , R. Gueta , R. Hagedorn , A. Losi , W. Gärtner , L. Petereit , M. Efetova , M. Schwarzel , T. G. Oertner , G. Nagel , P. Hegemann , J. Biol. Chem. 2011, 286, 1181. 21030594 10.1074/jbc.M110.185496 PMC3020725

[60] M. Folcher , S. Oesterle , K. Zwicky , T. Thekkottil , J. Heymoz , M. Hohmann , M. Christen , M. Daoud El‐Baba , P. Buchmann , M. Fussenegger , Nat. Commun. 2014, 5, 5392. 25386727 10.1038/ncomms6392 PMC4241983

[61] D. Tischer , O. D. Weiner , Nat. Rev. Mol. Cell Biol. 2014, 15, 551. 25027655 10.1038/nrm3837 PMC4145075

[62] X. Wang , X. Chen , Y. Yang , Nat. Methods 2012, 9, 266. 22327833 10.1038/nmeth.1892

[63] M. Grusch , K. Schelch , R. Riedler , E. Reichhart , C. Differ , W. Berger , A. Ingles‐Prieto , H. Janovjak , EMBO J. 2014, 33, 1713. 24986882 10.15252/embj.201387695 PMC4194103

[64] A. Pudasaini , K. K. El‐Arab , B. D. Zoltowski , Front. Mol. Biosci. 2015, 2, 18. 25988185 10.3389/fmolb.2015.00018 PMC4428443

[65] O. A. Sineshchekov , K.‐H. Jung , J. L. Spudich , Proc. Natl. Acad. Sci. USA 2002, 99, 8689. 12060707 10.1073/pnas.122243399 PMC124360

[66] P. Más , P. F. Devlin , S. Panda , S. A. Kay , Nature 2000, 408, 207. 11089975 10.1038/35041583

[67] L. J. Bugaj , A. T. Choksi , C. K. Mesuda , R. S. Kane , D. V Schaffer , Nat. Methods 2013, 10, 249. 23377377 10.1038/nmeth.2360

[68] K. Müller , R. Engesser , J. Timmer , F. Nagy , M. D. Zurbriggen , W. Weber , Chem. Commun. 2013, 49, 8970. 10.1039/c3cc45065a 23963496

[69] D. M. Shcherbakova , N. Cox Cammer , T. M. Huisman , V. V. Verkhusha , L. Hodgson , Nat. Chem. Biol. 2018, 14, 591. 29686359 10.1038/s41589-018-0044-1 PMC5964015

[70] T. A. Redchuk , A. A. Kaberniuk , V. V Verkhusha , Nat. Protoc. 2018, 13, 1121. 29700485 10.1038/nprot.2018.022 PMC6574219

[71] A. A. Kaberniuk , A. A. Shemetov , V. V Verkhusha , Nat. Methods 2016, 13, 591. 27159085 10.1038/nmeth.3864 PMC4927390

[72] R. P. Crefcoeur , R. Yin , R. Ulm , T. D. Halazonetis , Nat. Commun. 2013, 4, 1779. 23653191 10.1038/ncomms2800

[73] X. X. Zhou , H. K. Chung , A. J. Lam , M. Z. Lin , Science 2012, 338, 810. 23139335 10.1126/science.1226854 PMC3702057

[74] R. D. Airan , K. R. Thompson , L. E. Fenno , H. Bernstein , K. Deisseroth , Nature 2009, 458, 1025. 19295515 10.1038/nature07926

[75] M. van Wyk , J. Pielecka‐Fortuna , S. Löwel , S. Kleinlogel , PLoS Biol. 2015, 13, e1002143. 25950461 10.1371/journal.pbio.1002143 PMC4423780

[76] N. Kim , J. M. Kim , M. Lee , C. Y. Kim , K.‐Y. Chang , W. D. Heo , Chem. Biol. 2014, 21, 903. 24981772 10.1016/j.chembiol.2014.05.013

[77] A. Taslimi , J. D. Vrana , D. Chen , S. Borinskaya , B. J. Mayer , M. J. Kennedy , C. L. Tucker , Nat. Commun. 2014, 5, 4925. 25233328 10.1038/ncomms5925 PMC4170572

[78] H. Park , N. Y. Kim , S. Lee , N. Kim , J. Kim , W. Do Heo , Nat. Commun. 2017, 8, 30. 28646204 10.1038/s41467-017-00060-2 PMC5482817

[79] F. Kawano , H. Suzuki , A. Furuya , M. Sato , Nat. Commun. 2015, 6, 6256. 25708714 10.1038/ncomms7256

[80] S. Lyu , J. Fang , T. Duan , L. Fu , J. Liu , H. Li , Chem. Commun. 2017, 53, 13375. 10.1039/c7cc06991j 29200218

[81] E. Reichhart , A. Ingles‐Prieto , A.‐M. Tichy , C. McKenzie , H. Janovjak , Angew. Chem., Int. Ed. 2016, 55, 6339. 10.1002/anie.201601736 27101018

[82] S. Konermann , M. D. Brigham , A. E. Trevino , P. D. Hsu , M. Heidenreich , L. Le Cong , R. J. Platt , D. A. Scott , G. M. Church , F. Zhang , Nature 2013, 500, 472. 23877069 10.1038/nature12466 PMC3856241

[83] M. Endo , T. Ozawa , J. Photochem. Photobiol., C 2017, 30, 10.

[84] Y. I. Wu , D. Frey , O. I. Lungu , A. Jaehrig , I. Schlichting , B. Kuhlman , K. M. Hahn , Nature 2009, 461, 104. 19693014 10.1038/nature08241 PMC2766670

[85] H. Yumerefendi , D. J. Dickinson , H. Wang , S. P. Zimmerman , J. E. Bear , B. Goldstein , K. Hahn , B. Kuhlman , PLoS One 2015, 10, e0128443. 26083500 10.1371/journal.pone.0128443 PMC4471001

[86] D. Niopek , D. Benzinger , J. Roensch , T. Draebing , P. Wehler , R. Eils , B. Di Ventura , Nat. Commun. 2014, 5, 4404. 25019686 10.1038/ncomms5404 PMC4104460

[87] J. E. Toettcher , D. Gong , W. A. Lim , O. D. Weiner , Nat. Methods 2011, 8, 837. 21909100 10.1038/nmeth.1700 PMC3184382

[88] A. T. Vaidya , C.‐H. Chen , J. C. Dunlap , J. J. Loros , B. R. Crane , Sci. Signaling 2011, 4, ra50. 10.1126/scisignal.2001945 PMC3401549 21868352

[89] D. Chen , E. S. Gibson , M. J. Kennedy , J. Cell Biol. 2013, 201, 631. 23671313 10.1083/jcb.201210119 PMC3653365

[90] H. Wang , M. Vilela , A. Winkler , M. Tarnawski , I. Schlichting , H. Yumerefendi , B. Kuhlman , R. Liu , G. Danuser , K. M. Hahn , Nat. Methods 2016, 13, 755. 27427858 10.1038/nmeth.3926 PMC5137947

[91] M. Yazawa , A. M. Sadaghiani , B. Hsueh , R. E. Dolmetsch , Nat. Biotechnol. 2009, 27, 941. 19801976 10.1038/nbt.1569

[92] D. Strickland , Y. Lin , E. Wagner , C. M. Hope , J. Zayner , C. Antoniou , T. R. Sosnick , E. L. Weiss , M. Glotzer , Nat. Methods 2012, 9, 379. 22388287 10.1038/nmeth.1904 PMC3444151

[93] K. M. Bonger , R. Rakhit , A. Y. Payumo , J. K. Chen , T. J. Wandless , ACS Chem. Biol. 2014, 9, 111. 24180414 10.1021/cb400755b PMC3906921

[94] J. E. Toettcher , O. D. Weiner , W. A. Lim , Cell 2013, 155, 1422. 24315106 10.1016/j.cell.2013.11.004 PMC3925772

[95] T. Kakumoto , T. Nakata , PLoS One 2013, 8, e70861. 23951027 10.1371/journal.pone.0070861 PMC3737352

[96] A. Levskaya , O. D. Weiner , W. A. Lim , C. A. Voigt , Nature 2009, 461, 997. 19749742 10.1038/nature08446 PMC2989900

[97] E. J. Gomez , K. Gerhardt , J. Judd , J. J. Tabor , J. Suh , ACS Nano 2016, 10, 225. 26618393 10.1021/acsnano.5b05558

[98] L. J. Bugaj , D. P. Spelke , C. K. Mesuda , M. Varedi , R. S. Kane , D. V. Schaffer , Nat. Commun. 2015, 6, 6898. 25902152 10.1038/ncomms7898 PMC4408875

[99] S. Lee , H. Park , T. Kyung , N. Y. Kim , S. Kim , J. Kim , W. Do Heo , Nat. Methods 2014, 11, 633. 24793453 10.1038/nmeth.2940

[100] E. Mills , X. Chen , E. Pham , S. Wong , K. Truong , ACS Synth. Biol. 2012, 1, 75. 23651071 10.1021/sb200008j

[101] A. D. Smart , R. A. Pache , N. D. Thomsen , T. Kortemme , G. W. Davis , J. A. Wells , Proc. Natl. Acad. Sci. USA 2017, 114, E8174. 28893998 10.1073/pnas.1705064114 PMC5625904

[102] N. Fukuda , T. Matsuda , T. Nagai , ACS Chem. Biol. 2014, 9, 1197. 24625002 10.1021/cb400849n

[103] T. Kyung , S. Lee , J. E. Kim , T. Cho , H. Park , Y.‐M. Jeong , D. Kim , A. Shin , S. Kim , J. Baek , J. Kim , N. Y. Kim , D. Woo , S. Chae , C.‐H. Kim , H.‐S. Shin , Y.‐M. Han , D. Kim , W. Do Heo , Nat. Biotechnol. 2015, 33, 1092. 26368050 10.1038/nbt.3350

[104] Y. Zhang , L. Huang , Z. Li , G. Ma , Y. Zhou , G. Han , ACS Nano 2016, 10, 3881. 27077481 10.1021/acsnano.6b02284 PMC4913700

[105] L. He , Y. Zhang , G. Ma , P. Tan , Z. Li , S. Zang , X. Wu , J. Jing , S. Fang , L. Zhou , Y. Wang , Y. Huang , P. G. Hogan , G. Han , Y. Zhou , eLife 2015, 4, e10024. 26646180 10.7554/eLife.10024 PMC4737651

[106] G. Ma , S. Wen , L. He , Y. Huang , Y. Wang , Y. Zhou , Cell Calcium 2017, 64, 36. 28104276 10.1016/j.ceca.2017.01.004 PMC5457325

[107] F. A. Ran , P. D. Hsu , J. Wright , V. Agarwala , D. A. Scott , F. Zhang , Nat. Protoc. 2013, 8, 2281. 24157548 10.1038/nprot.2013.143 PMC3969860

[108] A. A. Dominguez , W. A. Lim , L. S. Qi , Nat. Rev. Mol. Cell Biol. 2016, 17, 5. 26670017 10.1038/nrm.2015.2 PMC4922510

[109] D. B. T. Cox , R. J. Platt , F. Zhang , Nat. Med. 2015, 21, 121. 25654603 10.1038/nm.3793 PMC4492683

[110] Y. Nihongaki , F. Kawano , T. Nakajima , M. Sato , Nat. Biotechnol. 2015, 33, 755. 26076431 10.1038/nbt.3245

[111] X. X. Zhou , X. Zou , H. K. Chung , Y. Gao , Y. Liu , L. S. Qi , M. Z. Lin , ACS Chem. Biol. 2018, 13, 443. 28938067 10.1021/acschembio.7b00603 PMC5820652

[112] Y. Nihongaki , S. Yamamoto , F. Kawano , H. Suzuki , M. Sato , Chem. Biol. 2015, 22, 169. 25619936 10.1016/j.chembiol.2014.12.011

[113] L. R. Polstein , C. A. Gersbach , Nat. Chem. Biol. 2015, 11, 198. 25664691 10.1038/nchembio.1753 PMC4412021

[114] M. Park , A. J. Keung , A. S. Khalil , Genome Biol. 2016, 17, 183. 27582168 10.1186/s13059-016-1046-5 PMC5006378

[115] H. Zhang , A. E. Cohen , Trends Biotechnol. 2017, 35, 625. 28552428 10.1016/j.tibtech.2017.04.002 PMC5495001

[116] Á. Inglés‐Prieto , E. Reichhart , M. K. Muellner , M. Nowak , S. M. B. Nijman , M. Grusch , H. Janovjak , Nat. Chem. Biol. 2015, 11, 952. 26457372 10.1038/nchembio.1933 PMC4652335

[117] V. Busskamp , B. Roska , Curr. Opin. Neurobiol. 2011, 21, 942. 21708457 10.1016/j.conb.2011.06.001

[118] B. Y. Chow , E. S. Boyden , Sci. Transl. Med. 2013, 5, 177ps5. 10.1126/scitranslmed.3003101 23515075

[119] P. Tan , L. He , G. Han , Y. Zhou , Trends Biotechnol. 2017, 35, 215. 27692897 10.1016/j.tibtech.2016.09.002 PMC5316489

[120] Y. Xu , Y.‐M. Hyun , K. Lim , H. Lee , R. J. Cummings , S. A. Gerber , S. Bae , T. Y. Cho , E. M. Lord , M. Kim , Proc. Natl. Acad. Sci. USA 2014, 111, 6371. 24733886 10.1073/pnas.1319296111 PMC4035914

[121] M. Xie , M. Fussenegger , Biotechnol. J. 2015, 10, 10051018. 10.1002/biot.201400642 26010998

[122] T. Kim , M. Folcher , M. D.‐E. Baba , M. Fussenegger , Angew. Chem., Int. Ed. 2015, 54, 5933. 10.1002/anie.201412204 25788334

[123] Y. Wang , M. Wang , K. Dong , H. Ye , Biotechnol. J. 2018, 13, 1700160. 10.1002/biot.201700160 29144600

[124] J. Dumont , D. Euwart , B. Mei , S. Estes , R. Kshirsagar , Crit. Rev. Biotechnol. 2016, 36, 1110. 26383226 10.3109/07388551.2015.1084266 PMC5152558

[125] R. M. Hernández , G. Orive , A. Murua , J. L. Pedraz , Adv. Drug Delivery Rev. 2010, 62, 711. 10.1016/j.addr.2010.02.004 20153388

[126] B. Zhou , B. Shi , D. Jin , X. Liu , Nat. Nanotechnol. 2015, 10, 924. 26530022 10.1038/nnano.2015.251

[127] S. Shah , J.‐J. Liu , N. Pasquale , J. Lai , H. McGowan , Z. P. Pang , K.‐B. Lee , Nanoscale 2015, 7, 16571. 26415758 10.1039/c5nr03411f PMC4712042

[128] S. Il Park , D. S. Brenner , G. Shin , C. D. Morgan , B. A. Copits , H. U. Chung , M. Y. Pullen , K. N. Noh , S. Davidson , S. J. Oh , J. Yoon , K.‐I. Jang , V. K. Samineni , M. Norman , J. G. Grajales‐Reyes , S. K. Vogt , S. S. Sundaram , K. M. Wilson , J. S. Ha , R. Xu , T. Pan , T. Kim , Y. Huang , M. C. Montana , J. P. Golden , M. R. Bruchas , R. W. Gereau , J. A. Rogers , Nat. Biotechnol. 2015, 33, 1280. 26551059 10.1038/nbt.3415 PMC4880021

[129] J. G. McCall , T. Kim , G. Shin , X. Huang , Y. H. Jung , R. Al‐Hasani , F. G. Omenetto , M. R. Bruchas , J. A. Rogers , Nat. Protoc. 2013, 8, 2413. 24202555 10.1038/nprot.2013.158 PMC4005292

[130] T.‐i. Kim , J. G. McCall , Y. H. Jung , X. Huang , E. R. Siuda , Y. Li , J. Song , Y. M. Song , H. A. Pao , R.‐H. Kim , C. Lu , S. D. Lee , I.‐S. Song , G. Shin , R. Al‐Hasani , S. Kim , M. P. Tan , Y. Huang , F. G. Omenetto , J. A. Rogers , M. R. Bruchas , Science 2013, 340, 211. 23580530 10.1126/science.1232437 PMC3769938

[131] K. Berglund , K. Clissold , H. E. Li , L. Wen , S. Y. Park , J. Gleixner , M. E. Klein , D. Lu , J. W. Barter , M. A. Rossi , G. J. Augustine , H. H. Yin , U. Hochgeschwender , Proc. Natl. Acad. Sci. USA 2016, 113, E358. 26733686 10.1073/pnas.1510899113 PMC4725499

[132] K. Deisseroth , Nat. Methods 2011, 8, 26. 21191368 10.1038/nmeth.f.324 PMC6814250

[133] K. T. Roybal , W. A. Lim , Annu. Rev. Immunol. 2017, 35, 229. 28446063 10.1146/annurev-immunol-051116-052302 PMC5555230

[134] K. Müller , R. Engesser , S. Metzger , S. Schulz , M. M. Kämpf , M. Busacker , T. Steinberg , P. Tomakidi , M. Ehrbar , F. Nagy , J. Timmer , M. D. Zubriggen , W. Weber , Nucleic Acids Res. 2013, 41, e77. 23355611 10.1093/nar/gkt002 PMC3627562

[135] K. Müller , M. D. Zurbriggen , W. Weber , Nat. Protoc. 2014, 9, 622. 24556785 10.1038/nprot.2014.038

[136] E. Bender , Nature 2016, 540, S106. 28002399 10.1038/540S106a

